# Investigating the risk of metabolic and cardiovascular comorbidities among patients with parathyroid cancer: a nationwide representative cohort study in Taiwan

**DOI:** 10.1186/s12916-023-02946-z

**Published:** 2023-07-10

**Authors:** Ming-Chieh Tsai, Min-Shu Hsu, Hsin-Yin Hsu, Tzu-Lin Yeh, Chun-Ju Chiang, Wen-Chung Lee, Jing-Rong Jhuang, Shih-Ping Cheng, Po-Jung Tseng, Kuo-Liong Chien

**Affiliations:** 1 Institute of Epidemiology and Preventive Medicine, College of Public Health, National Taiwan University, Room 517, No.17, Xu-Zhou Rd., Taipei City, 10055 Taiwan; 2 Division of Endocrinology and Metabolism, Department of Internal Medicine, Taipei Mackay Memorial Hospital, Section 2, Zhongshan North Road, No. 92 Taipei City, 10449 Taiwan; 3 Department of Medicine, Mackay Medical College, No. 46, Sec. 3, Zhongzheng Rd., Sanzhi Dist., New Taipei City, 25245 Taiwan; 4 Department of Medical Research, Mackay Memorial Hospital, No. 45, Minsheng Rd., Tamsui Dist., New Taipei City, 251404 Taiwan; 5 Department of Family Medicine, Taipei MacKay Memorial Hospital, No. 92, Section 2, Zhongshan North Road, Taipei City, 10449 Taiwan; 6 Department of Family Medicine, East District, Hsinchu MacKay Memorial Hospital, No. 690, Section 2, Guangfu Road, Hsinchu City, 30071 Taiwan; 7 Taiwan Cancer Registry, No.17, Xu-Zhou Rd., Taipei, 10055 Taiwan; 8 Innovation and Policy Center for Population Health and Sustainable Environment, College of Public Health, National Taiwan University, No.17, Xu-Zhou Rd., Taipei, 10055 Taiwan; 9 Division of General Surgery, Department of Surgery, Taipei Mackay Memorial Hospital, No. 92, Section 2, Zhongshan North Road, Taipei City, 10449 Taiwan; 10 Division of Cardiovascular Surgery, Department of Surgery, Hsin Chu Armed Force Hospital, No. 3, Wuling Rd., North Dist., Hsinchu City, 300 Taiwan; 11 Department of Internal Medicine, National Taiwan University Hospital, No. 7, Zhongshan S. Rd., Zhongzheng Dist., Taipei City, Taiwan; 12 Population Health Research Center, National Taiwan University Hospital, Taipei, Taiwan

**Keywords:** Parathyroid cancer, Cardiovascular disease, Diabetes mellitus

## Abstract

**Background:**

This study aimed to determine whether primary parathyroid cancer patients were associated with increased metabolic and cardiovascular comorbidities in comparison to the general population.

**Methods:**

We used the National Taiwan Cancer Registry Database to construct a cohort of patients with parathyroid cancer from January 1, 2004, to December 31, 2019. We compared the incidence of hypertension, diabetes mellitus, hyperlipidemia, atrial fibrillation, coronary heart disease, and heart failure with the general population matched based on a propensity score in a one-to-five fashion.

**Results:**

A total of 72 parathyroid cancer patients and 360 matched general population (mean age: 55 years; 59% women) were included, with different exclusive numbers for each metabolic and cardiovascular comorbidity cohort. The number of cases based on a total of 2347.7 person-years of observation included 53 deaths, 29 hypertension, 9 diabetes, 13 hyperlipidemia, 10 atrial fibrillation, 18 coronary artery disease, and 13 heart failure. According to multivariate analysis, parathyroid cancer remained significantly associated with diabetes [hazard ratio (HR): 9.28; 95% confidence interval (CI): 1.72–50.07], hyperlipidemia (HR: 5.86; 95% CI: 1.61–21.31), and heart failure (HR: 4.46; 95% CI: 1.18–16.84). Sub-distribution of competing mortality events and subgroup analysis showed robust evidence of metabolic and cardiovascular comorbidities. This national cohort study demonstrated that adult parathyroid cancer patients had a significantly higher incidence of diabetes mellitus, hyperlipidemia, and heart failure than the general population.

**Conclusions:**

An increased risk of metabolic and cardiac comorbidities among parathyroid cancer patients required great caution.

**Supplementary Information:**

The online version contains supplementary material available at 10.1186/s12916-023-02946-z.

## Background

Parathyroid cancer is a rare malignant endocrine neoplasm that occurs in one case per 10 million people in the USA [1] but has shown increasing incidence in the last few decades [2]. Parathyroid cancer accounts for 0.005% of all cancers and the 5-year and 10-year overall survival rates range from 81 to 92% and 49 to 77%, respectively [2–6]. Although patients usually undergo frequent surgical interventions for multiple recurrences, there is currently no universally recommended staging system, standardized treatment or management protocol for parathyroid cancer. There are several possible explanations for this situation. First, despite initial clinical assessment of rare parathyroid cancer, there may be insufficient evidence for further analysis. Second, most cases are confirmed only upon postoperative histological examination, as unclear pathophysiology limits preoperative and intraoperative diagnosis [7]. Third, in contrast to most cancers, survival is generally correlated with tumor size, lymph nodes, and distant metastasis; however, the morbidity and mortality rates from parathyroid cancer are usually a consequence of metabolic complications rather than tumor burden [8].

Patients with parathyroid cancer often have severe hyperparathyroidism, and elevated serum parathyroid hormone (PTH) levels (> tenfold) [9, 10], which leads to marked hypercalcemia and clinical symptoms such as fatigue, bone complications, renal manifestations, and cardiac arrhythmia [11]. In addition to the typical target organs regulated by PTH, the links between metabolic and cardiac comorbidities and hyperparathyroidism are conflicting. Research has revealed that primary hyperparathyroidism may be associated with higher insulin resistance [12], elevated blood pressure [13], increased body weight [14], abnormal lipid profile, severe coronary artery calcification score, and myocardial infarction [15, 16], while other analyses refute the association between hyperparathyroidism and metabolic and cardiovascular diseases [17–20]. Previous research has shown that primary hyperparathyroidism may be associated with hypertension, diabetes, and cardiovascular disease. Compared to the general population, patients with primary hyperparathyroidism were 3.1 and 2.64 times more likely to develop hypertension and diabetes, respectively [21].

Hypercalcemia and insulin resistance are known contributors to metabolic and cardiovascular diseases. Considering the rarity of parathyroid cancer cases and its poor prognosis as a result of its concomitant association with hypercalcemia, there remains a lack of evidence on the long-term metabolic and cardiovascular consequences of parathyroid cancer which requires further attention. Hence, this nationwide representative cohort study aimed to assess the link between parathyroid cancer and hypertension, diabetes, and hyperlipidemia risk and evaluate chronic metabolic disease persistence after adjusting for competing mortality events.

## Methods

### Study population and design

The National Taiwan Cancer Registry Database (NTCRD), founded in 1979, is a population-based retrospective cohort study based on a 97.8% representative sample of Taiwanese cancer patients. Details of the NTCRD, its methods, and participants have been previously reported [22]. The NTCRD has undergone three revisions since inception and has merged with the National Health Insurance Research Database (NHIRD) and national death records. We used person-level longitudinal NHIRD registration and claims data between January 1, 2004, and December 31, 2019.

### Exposure

Patients were included in the study based on the diagnosis of parathyroid cancer (ICD-O-FT: 1941 during 2007–2013; ICD-0–3 C750 during 2013–2018) from January 1, 2007, to December 31, 2018. We excluded patients younger than 20 years of age, those who were diagnosed before January 1, 2007, and with missing age or sex demographic information (Additional file 1: Fig. S1). Individuals were matched based on variables including age, sex, urbanization, occupation, and index year, completed in a one-to-five fashion. For the limited sample size in our database, a total of 72 patients with parathyroid cancer and 360 sex-and age-matched general population, we build six cohort according to various outcome assessments to reduce the case number wasting. In each cohort, we enrolled participants with and without parathyroid cancer and excluded pre-existing comorbidities according to different ascertainment.

### Outcomes

The primary outcomes included one hospitalization with the first to fifth diagnosis of hypertension, diabetes mellitus, or hyperlipidemia, or one outpatient clinic with combined anti-hypertension, anti-diabetes, or lipid-lowering medications at least 7 days after the index date. Additionally, primary outcomes included atrial fibrillation, coronary artery disease, and heart failure according to once hospitalization or twice outpatient clinical diagnoses. The secondary outcomes were overall and cancer-specific mortalities. Study protocols and consent forms were approved by the institutional review board at Mackay Memorial Hospital. The requirement for informed consent was waived due to de-identified data.

### Statistical analysis

NTCRD were analyzed from January 1, 2007, to December 31, 2018, and linked with NHIRD from January 1, 2004, to December 31, 2019. We compared patients with primary parathyroid cancer with age, sex, occupation, geographic, and index year matched to the general population during follow-up using two-tailed *t*-tests for continuous variables and *χ* ^2^ tests for categorical baseline variables. Baseline characteristics during the pre-index period were reported as proportions for categorical variables and as means with standard deviation (SD) for continuous variables. Survival probabilities were estimated using the Kaplan–Meier method, and the log-rank test was analyzed. As parathyroid cancer patients are at a higher risk of mortality than non-parathyroid cancer patients, the cumulative incidence function was plotted based on the cumulative incidence competing risk analysis.

We performed unadjusted and multivariable Cox proportional hazards regression to estimate hazard ratios (HRs) and 95% confidence intervals (CIs) with model 1 adjusted for age and sex reducing the incomplete-matching bias. Further models were adjusted for each outcome, including occupation (white collar, blue collar, and other), urbanization, average monthly income (< 35,000 NTD, ≥ 35,000 NTD), chronic kidney disease, nephrolithiasis, and osteoporosis. The fulfillment of proportional hazards assumptions for the Cox model was checked using standard methods, log (-log (survival function)). In addition, competing risk regression analyses with the sub-distribution hazard model (Fine and Gray model) were conducted using competing mortality.

To assess the risk of metabolic and cardiovascular comorbidity progression, the incidence estimated in different years since the parathyroid cancer diagnosis was stratified by outcome. To explore whether there were contrasting finding within the parathyroid cancer patients and general population, we performed subgroup analyses assessing the metabolic and cardiovascular comorbidities among patients stratified on age criteria (< 60 years vs ≥ 60 years) and interaction products with unadjusted and multivariable-adjusted models. A likelihood ratio test of the interaction term was used to determine the modification of the association. We plotted the progression of hazard ratio for all metabolic and cardiovascular comorbidities.

A sensitivity analysis was performed to assess the metabolic and cardiovascular comorbidities among parathyroid cancer patients compared with the all-covariate-matched general population, including original age, sex, urbanization, occupation, and index year in model and additionally average monthly income (0–34,999, ≥ 35,000 NTD), chronic kidney disease (baseline present and absence), nephrolithiasis (baseline present and absence), and osteoporosis (baseline present and absence). All analyses were conducted with SAS, version 9.4 (SAS Institute Inc., NC, USA) and STATA version 16 (StataCorp LLC, TX, USA), using a 2-tailed test. Statistical significance level was set at *p* < 0.05.

## Results

**Fig.1:**
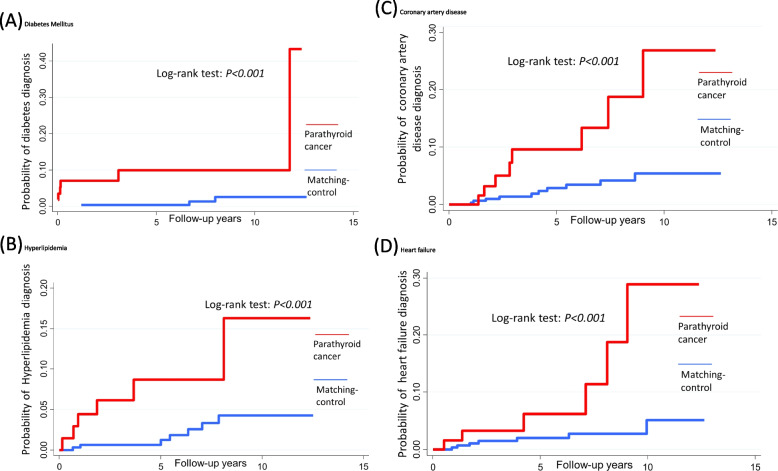
**A**–**D** Kaplan–Meier curves of diabetes mellitus, hyperlipidemia, coronary artery disease, and heart failure cases between parathyroid cancer patients compared with general population

A total of 72 patients with parathyroid cancer and 360 sex- and age-matched general population ≥ 20 years of age who were enrolled in the National Taiwan Cancer Registry Database cohort from 2007 to 2019 participated in the present study. According to various primary outcome assessments, both the enrolled cohort with and without parathyroid cancer had exclusive pre-existing comorbidities. We included the study population with no past specific comorbidities for each outcome. The final number of patients with and without parathyroid cancer in the hypertension, diabetes, hyperlipidemia, atrial fibrillation, coronary artery disease, and heart failure cohorts were 45 and 340, 58 and 311, 68 and 330, 70 and 344, 69 and 335, and 64 and 307, respectively (Fig. 1).

Considering the interesting outcome of heart failure, among the 64 primary parathyroid cancer patients and 307 individuals of the general population without primary parathyroid cancer, the mean (SD) age at the index date was 55.6 (13.3) and 55 (13.1) years old, 38 (59.4%) and 181 (59%) were women, and 42 (65.6%) and 222 (72.3%) resided in urban areas (Table 1). Patients with parathyroid cancer were more likely to have chronic kidney disease, nephrolithiasis, and osteoporosis (Additional file 1: Table S1).

**Table 1:** Comparison of baseline characteristics between adult parathyroid cancer patients and matching control group exclusive preexisting heart failure cohort

Baseline Characteristics of adult parathyroid carcinoma and matching controls			
	Patients (*n* = 64)	Control group (*n* = 307)	*P* value
Women, *n* (%)	38 (59.4)	181 (59)	0.95
Age:			
20–39 years old, *n* (%)	8 (12.5)	40 (13)	0.96
40–59 years old, *n* (%)	41 (64.1)	200 (65.2)	
≥ 60 years old, *n* (%)	15 (23.4)	67 (21.8)	
Mean age, years old (SD)	55.6 (13.3)	55 (13.1)	0.77
Follow-up duration, mean years (SD)	4.8 (3.1)	5.8 (3.4)	0.03
Occupation			
White collar, *n* (%)	27 (42.2)	141 (45.9)	0.66
Blue collar, *n* (%)	27 (42.2)	111 (36.2)	
Other, *n* (%)	10 (15.6)	55 (17.9)	
Urbanization (%)	42 (65.6)	222 (72.3)	0.28
Non-urbanization (%)	22 (34.4)	85 (27.7)	
Month income			
0–35,000 NTD, *n* (%)	54 (84.4)	247 (80.5)	0.47
≥ 35,000 NTD, *n* (%)	10 (15.6)	60 (19.5)	
Chronic kidney disease, *n* (%)	44 (68.8)	38 (12.4)	< 0.001
Nephrolithiasis, *n* (%)	22 (34.4)	18 (5.9)	< 0.001
Osteoporosis, *n* (%)	7 (10.9)	13 (4.2)	0.031

### Association of primary parathyroid cancer and the risk of metabolic and cardiovascular comorbidities

Figure 1 and Fig. S2 show the Kaplan–Meier curve for the incidence of each outcome during the mean 4.8 years of follow-up. Parathyroid cancer patients had a higher incidence than those with no parathyroid cancer diagnosis (Table 2) for diabetes mellitus (*p* < 0.001), hyperlipidemia (*p* = 0.002), atrial fibrillation (*p* = 0.034), coronary artery disease (*p* < 0.001), and heart failure (*p* = 0.002). Cumulative incidence plots after adjusting for competing mortality are shown in Fig. S3.

**Table 2:** The incidence case, follow-up person-years and rate of individual hypertension, diabetes mellitus, hyperlipidemia, atrial fibrillation, coronary artery disease and heart failure comorbidities

	Hypertension			Diabetes			Hyperlipidemia		
	Control	PTC	Log-rank	Control	PTC	Log-rank	Control	PTC	Log-rank
*N*	206	45		262	58		330	68	
Event	23	6		3	6		7	6	
Person-year	1113	207		1499	284.3		1832	323.2	
Incidence rate (/1000 person-year)	20.7	29	0.44	2	21.1	< 0.001	3.8	18.6	0.002
Unadjusted	1	1.43 (0.58–3.51)		1	10.07 (2.51–40.41)		1	4.78 (1.6–14.27)	
Model 1	1	1.52 (0.48–4.84)		1	15.18 (2.48–92.98)		1	8.35 (2.1–33.25)	
Model 2	1	1.5 (0.6–3.73)		1	10.55 (2.59–43.02)		1	4.7 (1.57–14.07)	
Model 3	1	1.12 (0.37–3.41)		1	9.28 (1.72–50.07)		1	5.86 (1.61–21.31)	
	Atrial fibrillation			Coronary artery disease			Heart failure		
	Control	PTC	Log-rank	Control	PTC	Log-rank	Control	PTC	Log-rank
*N*	344	70		335	69		307	64	
Event	6	4		10	8		7	6	
Person-year	1919	325.1		1868	333.8		1716	307.7	
Incidence rate (/1000 person-year)	3.1	12.3	0.034	5.4	24	< 0.001	4.1	19.5	0.002
Unadjusted	1	3.6 (1.01–12.78)		1	4.51 (1.78–11.46)		1	4.98 (1.66–14.92)	
Model 1	1	3.81 (1.06–13.73)		1	4.44 (1.73–11.42)		1	4.89 (1.6–14.97)	
Model 2	1	3.38 (0.95–12.05)		1	4.74 (1.86–12.13)		1	5.28 (1.71–16.33)	
Model 3	1	2.09 (0.46–9.59)		1	1.69 (0.5–5.63)		1	4.46 (1.18–16.84)	

Model 1: adjusted for age and sex; Model 2: adjusted occupation, urbanization, average month income; Model 3: Model 2 additionally chronic kidney disease, nephrolithiasis, osteoporosis

*PTC* parathyroid cancer

**Fig. 2:**
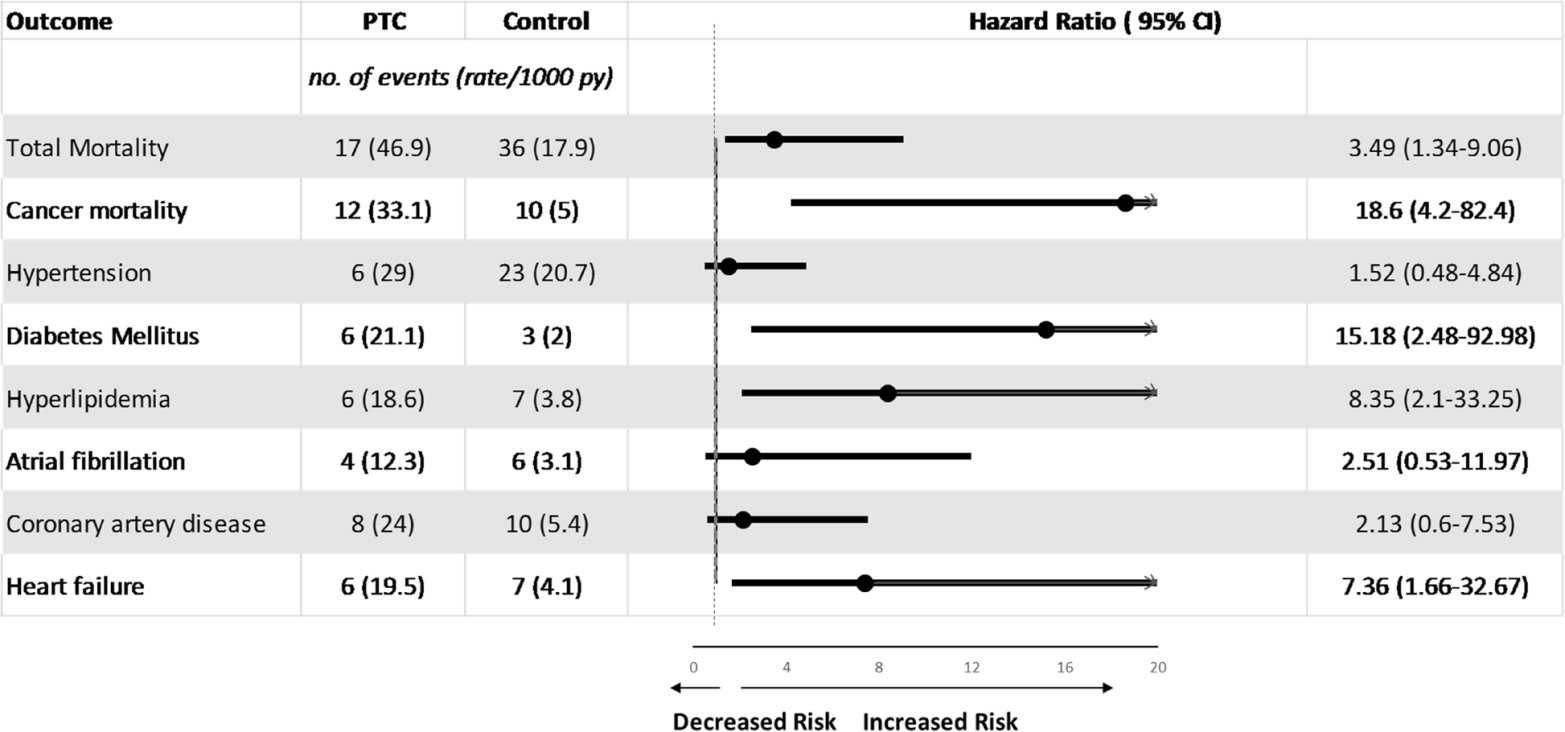
Multivariable-adjusted hazard ratios with 95% confidence interval of all cause and cancer-specific mortality, hypertension, diabetes, hyperlipidemia, atrial fibrillation, coronary artery disease, and heart failure among parathyroid cancer patients compared with matching population

The univariate and multivariate analyses of metabolic and cardiovascular comorbidities are presented in Table 2. After adjusting for personal characteristics and other chronic diseases, individuals with parathyroid cancer showed 9.28 (95% CI: 1.72–50.07), 5.86 (95% CI: 1.61–21.31), and 4.46 (95% CI: 1.18–16.84) independent odds of new-onset diabetes, hyperlipidemia, and heart failure, respectively, compared to those without parathyroid cancer. Moreover, parathyroid cancer was associated with an increased risk of atrial fibrillation (HR: 3.81; 95% CI: 1.06–13.73) and coronary artery disease (HR: 4.74; 95% CI: 1.86–12.13) in partially adjusted models. Nevertheless, endocrinologic cancer was no longer significantly associated with either atrial fibrillation (HR: 2.09; 95% CI: 0.46–9.59) or coronary artery disease (HR: 1.69; 95% CI: 0.5–5.63) after adjustment for chronic kidney disease, nephrolithiasis, and osteoporosis (Fig. 2).

**Fig. 3:**
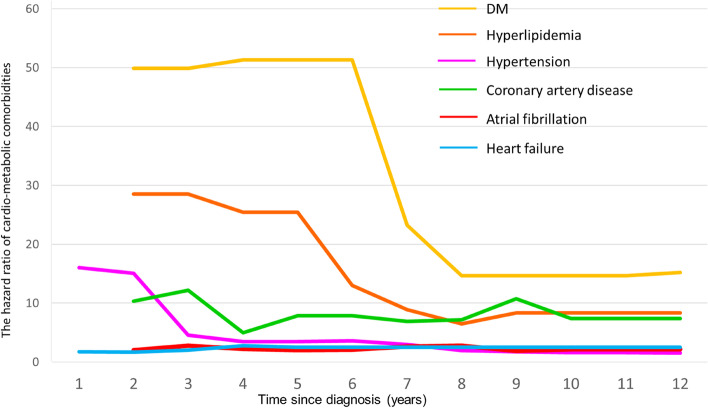
Estimated the Cox hazard ratios of parathyroid cancer with cardio-metabolic comorbidities stratified by time since diagnosis (years)

Likewise, the risk for total and cancer-specific mortality was 3.49 (95% CI: 1.34–9.06) and 18.6 (95% CI: 4.2–82.4) times significantly higher among parathyroid cancer patients. Furthermore, the sub-distribution hazard ratios demonstrated robust evidence after competing mortality events, in which the incidental risk for diabetes mellitus, hyperlipidemia, and heart failure significantly increased (Table S3). Figure 3 reveals that the highest odds among parathyroid cancer patients appeared within 6 years and dramatically dropped after the sixth year. However, the long-term trend for an association between parathyroid cancer and comorbidities persisted for decades (Fig. 3 and Table S4).

Overall, the parathyroid cancer patients had higher cardiometabolic incidence among both age < 60 or ≥ 60 years subgroup although the coronary artery disease risk was lower among patients younger than 60 years old (HR, 0.33; 95% CI, 0.02–6.51) (*P* = 0.012 for interaction) (Fig.S4 and table S5). A sensitivity analysis was done and compatible with previous demonstration (Table S6). The odds of metabolic and cardiovascular comorbidities among parathyroid cancer patients were robust (Table S7).

## Discussion

Our main finding from this 12-year observation of a national parathyroid cancer cohort was that parathyroid cancer was significantly associated with an increased risk of diabetes mellitus, hyperlipidemia, and heart failure, independent of several chronic diseases adjusted and death competing. Using Cox regression stratified by the years since cancer diagnosis, our findings demonstrated that the highest odds appeared within 6 years, and the trend persisted for decades.

To our knowledge, our study is the first national population-based analysis to demonstrate evidence of an association between parathyroid cancer and metabolic and cardiovascular comorbidities. Although no previous studies have examined the association among parathyroid cancer patients, the results are consistent with prior investigations of the increased incidence of diabetes, metabolic syndrome, obesity, and coronary artery disease among individuals with primary hyperparathyroidism [23]. Observational studies have reported a 38.9% increase in the prevalence of insulin resistance [12] and an 8–59% increase in metabolic syndrome [16, 24] in patients with primary hyperparathyroidism, compared to the general population. Furthermore, other data including systolic blood pressure, 24-h pulse wave velocity, 5.1-fold positive coronary artery calcification score [25], and left ventricular mass index were associated with serum PTH level in patients with primary hyperparathyroidism [26]. Current studies also demonstrated a significant likelihood of cardiovascular risk with a 3.5-fold odds ratio of coronary artery disease [12] and stroke in patients with primary hyperparathyroidism compared to the general population. Our findings confirmed the association between high PTH serum levels and the consequences of metabolic and cardiac disorders. In contrast, previous studies have shown an elevated prevalence of hypertension among patients with primary hyperparathyroidism (OR: 1.2–1.5, *p* value < 0.0001) as compared to the general population [23], although this association was not observed in our study. It is possible that the sample size of our parathyroid cancer cohort was too small to demonstrate a significant difference. Association between parathyroid cancer and coronary artery disease risk was difference by age subgroup. However, wider confidence intervals in relation to the estimate coronary artery disease might indicate the association instability and further research would be warrant.

The role of parathyroid hormone levels in modulating human metabolism has generated intense research interest, denoted by the plethora of mechanisms that underpin various epidemiologic studies. For example, one study reported that hyperfunction of PTH induces reduction of insulin-stimulated glucose uptake by decreasing GLUT4 expression and suppressing insulin signaling [27]. A meta-analysis of 17 pooled studies showed that individuals with primary hyperparathyroidism were 3.34 kg heavier in terms of body weight (95% CI: 1.97–4.71) and had increased body mass index of 1.13 kg/m^2^ (95% CI: 0.28–2.55) compared to the general population [14]. Although the link between obesity and hyperparathyroidism is unclear, the current study hypothesized that excess PTH reduces the adipocyte lipolytic response to catecholamine by increasing calcium influx. Another epidemiological study demonstrated that elevated LDL/HDL ratio, elevated triglyceride levels, and low HDL levels were significantly associated with normo-calcemic hyperparathyroidism as compared to the general population [28].

Numerous factors might explain the alternative physiological effects on the heart by PTH. The PTH receptor is found in cardiac myocytes, vascular smooth cells, and endothelial cells. By direct vasorelaxant mechanism on vascular smooth muscle, PTH could induce direct vasodilation effects on vascular smooth cells [29]. Other studies have shown that PTH accelerates the formation of vascular atherosclerosis and remodeling by stimulating vascular growth factors [30]. Severe excess PTH results in left ventricular hypertrophy and remodeling by numerous factors, such as increasing vascular calcification, accelerating atherosclerosis, increasing chronotropy and hypertrophy of cardiomyocytes, and overt metabolic syndrome including insulin resistance, dyslipidemia, and overweight [31]. Consequently, both hypercalcemia and metabolic syndrome can lead to cardiovascular risk among patients with hyperparathyroidism. Additionally, the alternation of left ventricle mass thickening and arrhythmia triggered by PTH hyperfunction results in metabolic variables and cardiovascular risk as noted in epidemiological studies. This is consistent with our observation of significantly increasing long-term incidence of diabetes, hyperlipidemia, and heart failure.

Our results have important implications for current evidence regarding the clinical care of primary parathyroid cancer. From an endocrinological perspective, comprehensive consideration of metabolic and cardiac complications, not only skeletal comorbidity, may provide pragmatic benefits to parathyroid cancer patients and improve their long-term quality of life. Regarding the pathophysiological reasons for the higher standardized incidence rate of diabetes, hyperlipidemia, and heart failure among parathyroid cancer patients, further epidemiological studies on the association between PTH serum levels and insulin resistance, lipid profile, and severity of endothelial calcification should be conducted.

### Strengths and limitations

This study had several strengths. The current study represents the first evidence of long-term comorbidities among rare, primary parathyroid cancer survivors. Furthermore, in contrast to many previous primary parathyroid cancer cohorts from a single medical institution, our cohort was from a national database with comprehensive medical records before cancer diagnosis and long-term follow-up.

This study has several limitations. First, there is limited clinical, biochemical, and cancer information, including staging, first-time surgical treatment, chemotherapy, radiation use, hormone levels, body mass index, lifestyle habits, and race. However, according to comprehensive comorbidity information for both patients and the matching population from the national insurance database (which comprised 99% of citizens’ medical records), our studies corrected potential confounding factors to estimate more robust evidence. Second, the observational cohort study design of our research and the retrospective nature of data collection cannot be used to establish causality. Although our investigation was the first report, further research is still warranted. Third, there was a lack of sufficient evidence to support lead-time bias in our study. Compared to the general population, thyroid cancer patients seem to visit outpatient clinics more frequently for follow-up care, which may result in lead-time bias. However, this potential concern may be ameliorated due to the increasingly convenient and inexpensive medical health care available in Taiwan and the fact that similar medical resources are available for both parathyroid cancer patients and the general population. Finally, there were several outcome variables in our studies which might increase type 1 error. For the aim of our study investigating the cardio-metabolic hazard ratio among a rare disease, we classified the outcome into several diseases for more detailed evidence. Further research should be warrant for the causality.

## Conclusions

In summary, the findings of this national cohort study demonstrated that adult parathyroid cancer patients had a significantly higher incidence of diabetes mellitus, hyperlipidemia, and heart failure than the general population. Additionally, the evidence suggests that adult parathyroid cancer survivors experience long-term metabolic and cardiovascular comorbidities; thus, further research is warranted.

## Supplementary Information

**Additional file 1: Tables S1.** Baseline Characteristics. **Tables S2.** Cox proportional hazard regression of total and cancer-specific mortality. **Tables S3.** Estimated sub-distribution competing hazard ratios for metabolic and heart comorbidities and mortality. **Tables S4.** Estimated Cox proportional hazard ration with 95% confidence interval stratified by time since diagnosis. **Tables S5.** Metabolic and heart comorbidities stratified by age less than 60 years old or older than 60 years old. **Tables S6.** Sensitivity analysis (a) hypertension cohort (b) diabetes cohort (c) hyperlipidemia cohort (d) atrial fibrillation cohort (e) coronary artery disease (f) heart failure. **Tables S7.** The sensitivity analysis for all-covariate-matching parathyroid cancer population. **Figures S1.** Flowchart of Patients with parathyroid cancer. **Figures S2.** The Kaplan-Meier of overall survival, hypertension and atrial fibrillation. **Figures S3.** The cumulative incidence of metabolic and heart comorbiditiesHypertensiondiabetes mellitushyperlipidemiaatrial fibrillationcoronary artery diseaseheart failure. **Figures S4.** Association of adult parathyroid cancer stratified by age less than 60 years old or older than 60 years old. **Figures S5.** The log) versus loghypertensiondiabetes mellitushyperlipidemiaatrial fibrillationcoronary artery diseaseheart failure.

## Data Availability

The Taiwan Cancer Registry Center data are publicly available at https://twcr.tw/.

## Abbreviations

CIs: Confidence intervals
HRs: Hazard ratios
NHIRD: National Health Insurance Research Database
NTCRD: National Taiwan Cancer Registry Database
PTH: Parathyroid hormone
SD: Standard deviation

## References

[1] Lee PK, Jarosek SL, Virnig BA, Evasovich M, Tuttle TM (2007). Trends in the incidence and treatment of parathyroid cancer in the United States. Cancer.

[2] Sadler C, Gow KW, Beierle EA, Doski JJ, Langer M, Nuchtern JG, et al. Parathyroid carcinoma in more than 1,000 patients: a population-level analysis. Surgery. 2014;156(6):1622–9; discussion 9–30. 10.1016/j.surg.2014.08.069 25456964

[3] Hundahl SA, Fleming ID, Fremgen AM, Menck HR. Two hundred eighty-six cases of parathyroid carcinoma treated in the U.S. between 1985–1995: a National Cancer Data Base Report. The American College of Surgeons Commission on Cancer and the American Cancer Society. Cancer. 1999;86(3):538–44. 10.1002/(sici)1097-0142(19990801)86:3<538::aid-cncr25>3.0.co;2-k 10430265

[4] Asare EA, Sturgeon C, Winchester DJ, Liu L, Palis B, Perrier ND (2015). Parathyroid carcinoma: an update on treatment outcomes and prognostic factors from the National Cancer Data Base (NCDB). Ann Surg Oncol.

[5] Villar-del-Moral J, Jiménez-García A, Salvador-Egea P, Martos-Martínez JM, Nuño-Vázquez-Garza JM, Serradilla-Martín M (2014). Prognostic factors and staging systems in parathyroid cancer: a multicenter cohort study. Surgery.

[6] Limberg J, Stefanova D, Ullmann TM, Thiesmeyer JW, Bains S, Beninato T (2021). The use and benefit of adjuvant radiotherapy in parathyroid carcinoma: a national cancer database analysis. Ann Surg Oncol.

[7] Koea JB, Shaw JH (1999). Parathyroid cancer: biology and management. Surg Oncol.

[8] Owen RP, Silver CE, Pellitteri PK, Shaha AR, Devaney KO, Werner JA (2011). Parathyroid carcinoma: a review. Head Neck.

[9] Xue S, Chen H, Lv C, Shen X, Ding J, Liu J (2016). Preoperative diagnosis and prognosis in 40 parathyroid carcinoma patients. Clin Endocrinol (Oxf).

[10] Ryhänen EM, Leijon H, Metso S, Eloranta E, Korsoff P, Ahtiainen P (2017). A nationwide study on parathyroid carcinoma. Acta Oncol.

[11] Christakis I, Silva AM, Kwatampora LJ, Warneke CL, Clarke CN, Williams MD (2016). Oncologic progress for the treatment of parathyroid carcinoma is needed. J Surg Oncol.

[12] Luboshitzky R, Chertok-Schaham Y, Lavi I, Ishay A (2009). Cardiovascular risk factors in primary hyperparathyroidism. J Endocrinol Invest.

[13] Concistrè A, Grillo A, La Torre G, Carretta R, Fabris B, Petramala L (2018). Ambulatory blood pressure monitoring-derived short-term blood pressure variability in primary hyperparathyroidism. Endocrine.

[14] Bolland MJ, Grey AB, Gamble GD, Reid IR (2005). Association between primary hyperparathyroidism and increased body weight: a meta-analysis. J Clin Endocrinol Metab.

[15] Herrmann G, Hehrmann R, Scholz HC, Atkinson M, Lichtlen P, von zur Mühlen A, et al. Parathyroid hormone in coronary artery disease--results of a prospective study. J Endocrinol Invest. 1986;9(4):265–71. 10.1007/BF03346923 3782741

[16] Han D, Trooskin S, Wang X (2012). Prevalence of cardiovascular risk factors in male and female patients with primary hyperparathyroidism. J Endocrinol Invest.

[17] Tassone F, Gianotti L, Baffoni C, Cesario F, Magro G, Pellegrino M (2012). Prevalence and characteristics of metabolic syndrome in primary hyperparathyroidism. J Endocrinol Invest.

[18] Joborn H, Lundin L, Hvarfner A, Johansson G, Wide L, Ljunghall S (1989). Serum electrolytes and parathyroid hormone in patients in a coronary care unit. J Intern Med.

[19] Watson KE, Abrolat ML, Malone LL, Hoeg JM, Doherty T, Detrano R (1997). Active serum vitamin D levels are inversely correlated with coronary calcification. Circulation.

[20] Procopio M, Barale M, Bertaina S, Sigrist S, Mazzetti R, Loiacono M (2014). Cardiovascular risk and metabolic syndrome in primary hyperparathyroidism and their correlation to different clinical forms. Endocrine.

[21] Yu N, Donnan PT, Flynn RW, Murphy MJ, Smith D, Rudman A, et al. Increased mortality and morbidity in mild primary hyperparathyroid patients. The Parathyroid Epidemiology and Audit Research Study (PEARS). Clin Endocrinol (Oxf). 2010;73(1):30–4. 10.1111/j.1365-2265.2009.03766.x 20039887

[22] Yeh TL, Hsieh CT, Hsu HY, Tsai MC, Wang CC, Lin CY (2022). The risk of ischemic stroke in head and neck cancer patients and those who were treated with radiotherapy: a population-based cohort study. Cancer Epidemiol Biomarkers Prev.

[23] Kalla A, Krishnamoorthy P, Gopalakrishnan A, Garg J, Patel NC, Figueredo VM (2017). Primary hyperparathyroidism predicts hypertension: results from the National Inpatient Sample. Int J Cardiol.

[24] Mendoza-Zubieta V, Gonzalez-Villaseñor GA, Vargas-Ortega G, Gonzalez B, Ramirez-Renteria C, Mercado M (2015). High prevalence of metabolic syndrome in a mestizo group of adult patients with primary hyperparathyroidism (PHPT). BMC Endocr Disord.

[25] Koubaity O, Mandry D, Nguyen-Thi PL, Bihain F, Nomine-Criqui C, Demarquet L (2020). Coronary artery disease is more severe in patients with primary hyperparathyroidism. Surgery.

[26] Wetzel J, Pilz S, Grübler MR, Fahrleitner-Pammer A, Dimai HP, von Lewinski D (2017). Plasma parathyroid hormone and cardiovascular disease in treatment-naive patients with primary hyperparathyroidism: The EPATH trial. J Clin Hypertens (Greenwich).

[27] Chang E, Donkin SS, Teegarden D (2009). Parathyroid hormone suppresses insulin signaling in adipocytes. Mol Cell Endocrinol.

[28] Hagström E, Lundgren E, Rastad J, Hellman P (2006). Metabolic abnormalities in patients with normocalcemic hyperparathyroidism detected at a population-based screening. Eur J Endocrinol.

[29] Pang PK, Wang R, Shan J, Karpinski E, Benishin CG (1990). Specific inhibition of long-lasting, L-type calcium channels by synthetic parathyroid hormone. Proc Natl Acad Sci U S A.

[30] Rashid G, Bernheim J, Green J, Benchetrit S (2008). Parathyroid hormone stimulates the endothelial expression of vascular endothelial growth factor. Eur J Clin Invest.

[31] Pepe J, Cipriani C, Sonato C, Raimo O, Biamonte F, Minisola S (2017). Cardiovascular manifestations of primary hyperparathyroidism: a narrative review. Eur J Endocrinol.

