# Supplementary material for: Investigating the risk of metabolic and cardiovascular comorbidities among patients with parathyroid cancer: a nationwide representative cohort study in Taiwan
Source: BMC Med. 2023 Jul 10;21:249. doi: 10.1186/s12916-023-02946-z (PMC10332013; doi:10.1186/s12916-023-02946-z)

**Content**

[Supplemental Table 1. Baseline Characteristics of adult parathyroid carcinoma and matching controls without preexisting disease (a) hypertension cohort (b) diabetes cohort (c) hyperlipidemia cohort (d) atrial fibrillation cohort (e) coronary artery disease 3](#_Toc139432720)

[Supplemental Table 2. The all death and cancer specific death, follow-up person-years and mortality rate. Estimated cox proportional hazard regression and 95% confidence intervals specified results of total and cancer-specific mortality. 8](#_Toc139432721)

[Supplemental Table 3. Estimated sub-distribution competing hazard ratios for metabolic and heart comorbidities and mortality using multivariable regression model 9](#_Toc139432722)

[Supplemental table 4. Estimated Cox proportional hazard ration with 95% confidence interval stratified by time since diagnosis (years) 11](#_Toc139432723)

[Supplemental Table 5 Association of adult parathyroid cancer compared matching general population with metabolic and heart comorbidities stratified by age less than 60 years old or older than 60 years old 12](#_Toc139432724)

[Supplemental table 6. The baseline characteristics of adult parathyroid carcinoma and all-covariate-matched controls without preexisting disease in the sensitivity analysis (a) hypertension cohort (b) diabetes cohort (c) hyperlipidemia cohort (d) atrial fibrillation cohort (e) coronary artery disease (f) heart failure. 13](#_Toc139432725)

[Supplemental table 7. The incidence case, follow-up person-years and rate of individual metabolic and heart comorbidities and estimated cox proportional hazard regression and 95% confidence intervals specified results of hypertension, diabetes mellitus, hyperlipidemia, atrial fibrillation, coronary artery disease and heart failure in the sensitivity analysis for all-covariate-matching parathyroid cancer population. 19](#_Toc139432726)

[Supplement Figure 1, Flowchart of Patients with parathyroid cancer in National Taiwan Cancer Registry Database cohort from 2007 to 2018 who met Inclusion and Exclusion Criteria 21](#_Toc131510593)

[Supplement Figure 2. The Kaplan-Meier of overall survival, hypertension and atrial fibrillation cases among adult parathyroid cancer compared with matching general 22](#_Toc131510594)

[Supplement Figure 3. Estimated the cumulative incidence of metabolic and heart comorbidities among adult parathyroid cancer compared with general population. (a) Hypertension (b) diabetes mellitus (c) hyperlipidemia (e) atrial fibrillation (f) coronary artery disease (g) heart failure 25](#_Toc131510595)

[Supplement Figure 4. Association of adult parathyroid cancer compared matching general population with metabolic and heart comorbidities stratified by age less than 60 years old or older than 60 years old 31](#_Toc131510596)

[Supplement Figure 5. The log(-log(survival time)) versus log of metabolic and heart comorbidities event-free survival time including time independent covariates (A) hypertension (b)diabetes mellitus (c) hyperlipidemia (d) atrial fibrillation (e) coronary artery disease (f) heart failure 32](#_Toc131510597)

Supplemental Table 1. Baseline Characteristics of adult parathyroid carcinoma and matching controls without preexisting disease (a) hypertension cohort (b) diabetes cohort (c) hyperlipidemia cohort (d) atrial fibrillation cohort (e) coronary artery disease

(a) Hypertension

| Baseline Characteristics of adult parathyroid carcinoma and matching controls | | | |
| --- | --- | --- | --- |
|  | Patients (n=45) | Control group (n=206) | *P* value |
| Women, n (%) | 28 (62.2) | 132 (64.1) | 0.81 |
| Age: 20-39 years old, n (%) | 6 (13.3) | 30 (14.6) | 0.81 |
| 40-59 years old, n (%) | 30 (66.7) | 143 (69.4) |  |
| ≥60 years old, n (%) | 9 (20) | 33 (16) |  |
| Mean age, years old (SD) | 54.7 (13.2) | 53.4 (12.7) | 0.54 |
| Follow up duration, mean years (SD) | 4.6 (3.4) | 5.6 (3.3) | 0.07 |
| Occupation: White collar, n (%) | 22 (48.9) | 101 (49) | 0.36 |
| Blue collar, n (%) | 19 (42.2) | 71 (34.5) |  |
| Other, n (%) | 4 (8.9) | 34 (16.5) |  |
| Urbanization (%) | 27 (60) | 149 (72.3) | 0.1 |
| Non-urbanization (%) | 18 (40) | 57 (27.7) |  |
| Month Income: 0–35000 NTD, n (%) | 37 (82.2) | 158 (76.7) | 0.42 |
| ≥35000 NTD, n (%) | 8 (17.8) | 48 (23.3) |  |
| Chronic kidney disease, n (%) | 28 (62.2) | 22 (10.7) | <0.001 |
| Nephrolithiasis, n (%) | 14 (31.1) | 11 (5.3) | <0.001 |
| Osteoporosis, n (%) | 3 (6.6) | 11 (4.2) | 0.028 |

(b)Diabetes mellitus

| Baseline Characteristics of adult parathyroid carcinoma and matching controls | | | |
| --- | --- | --- | --- |
|  | Patients (n=58) | Control group (n=262) | *P* value |
| Women, n (%) | 33 (56.9) | 153 (58.4) | 0.83 |
| Age: 20-39 years old, n (%) | 8 (13.8) | 40 (15.3) | 0.73 |
| 40-59 years old, n (%) | 37 (63.8) | 175 (66.8) |  |
| ≥60 years old, n (%) | 13 (22.4) | 47 (17.9) |  |
| Mean age, years old (SD) | 55.4 (13.6) | 54.2 (13.3) | 0.53 |
| Follow up duration, mean years (SD) | 4.9 (3.5) | 5.9 (3.5) | 0.04 |
| Occupation: White collar, n (%) | 24 (41.4) | 126 (48.1) | 0.62 |
| Blue collar, n (%) | 23 (39.7) | 88 (33.6) |  |
| Other, n (%) | 11 (19) | 48 (18.3) |  |
| Urbanization (%) | 36 (62.1) | 188 (71.8) | 0.15 |
| Non-urbanization (%) | 22 (37.9) | 74 (28.2) |  |
| Month Income: 0–35000 NTD, n (%) | 49 (84.5) | 203 (77.5) | 0.24 |
| ≥35000 NTD, n (%) | 9 (15.5) | 59 (22.5) |  |
| Chronic kidney disease, n (%) | 38 (65.5) | 26 (9.9) | <0.001 |
| Nephrolithiasis, n (%) | 17 (29.3) | 16 (6.1) | <0.001 |
| Osteoporosis, n (%) | 4 (6.9) | 11 (4.2) | 0.38 |

(c) Hyperlipidemia

| Baseline Characteristics of adult parathyroid carcinoma and matching controls | | | |
| --- | --- | --- | --- |
|  | Patients (n=68) | Control group (n=330) | *P* value |
| Women, n (%) | 39 (57.4) | 191 (57.9) | 0.94 |
| Age: 20-39 years old, n (%) | 9 (13.2) | 45 (13.6) | 0.97 |
| 40-59 years old, n (%) | 43 (63.2) | 212 (64.2) |  |
| ≥60 years old, n (%) | 16 (23.5) | 73 (22.1) |  |
| Mean age, years old (SD) | 56 (13.7) | 55.7 (13.5) | 0.86 |
| Follow up duration, mean years (SD) | 4.8 (3.1) | 5.9 (3.3) | 0.01 |
| Occupation: White collar, n (%) | 29 (42.7) | 152 (46.1) | 0.76 |
| Blue collar, n (%) | 28 (41.2) | 120 (36.4) |  |
| Other, n (%) | 11 (16.2) | 58 (17.6) |  |
| Urbanization (%) | 42 (61.8) | 232 (70.3) | 0.17 |
| Non-urbanization (%) | 26 (38.2) | 98 (29.7) |  |
| Month Income: 0–35000 NTD, n (%) | 57 (83.8) | 264 (80) | 0.47 |
| ≥35000 NTD, n (%) | 11 (16.2) | 66 (20) |  |
| Chronic kidney disease, n (%) | 48 (70.6) | 42 (12.7) | <0.001 |
| Nephrolithiasis, n (%) | 23 (33.8) | 18 (5.5) | <0.001 |
| Osteoporosis, n (%) | 7 (10.3) | 19 (5.8) | 0.17 |

(d) Atrial fibrillation

| Baseline Characteristics of adult parathyroid carcinoma and matching controls | | | |
| --- | --- | --- | --- |
|  | Patients (n=70) | Control group (n=344) | *P* value |
| Women, n (%) | 41 (58.6) | 203 (59) | 0.95 |
| Age: 20-39 years old, n (%) | 9 (12.9) | 45 (13.1) | 0.98 |
| 40-59 years old, n (%) | 44 (62.9) | 219 (63.7) |  |
| ≥60 years old, n (%) | 17 (24.3) | 80 (23.3) |  |
| Mean age, years old (SD) | 56 (13.5) | 55.7 (13.4) | 0.87 |
| Follow up duration, mean years (SD) | 4.6 (3.2) | 5.9 (3.4) | 0.005 |
| Occupation: White collar, n (%) | 29 (41.4) | 155 (45.1) | 0.84 |
| Blue collar, n (%) | 27 (38.6) | 127 (36.9) |  |
| Other, n (%) | 14 (20) | 62 (18) |  |
| Urbanization (%) | 45 (64.3) | 242 (70.4) | 0.32 |
| Non-urbanization (%) | 25 (35.7) | 102 (29.7) |  |
| Month Income: 0–35000 NTD, n (%) | 59 (84.3) | 280 (81.4) | 0.57 |
| ≥35000 NTD, n (%) | 11 (15.7) | 64 (18.6) |  |
| Chronic kidney disease, n (%) | 49 (70) | 44 (12.8) | <0.001 |
| Nephrolithiasis, n (%) | 24 (34.3) | 18 (5.2) | <0.001 |
| Osteoporosis, n (%) | 7 (10) | 22 (6.4) | 0.28 |

(e) Coronary artery disease

| Baseline Characteristics of adult parathyroid carcinoma and matching controls | | | |
| --- | --- | --- | --- |
|  | Patients (n=69) | Control group (n=335) | *P* value |
| Women, n (%) | 40 (58) | 196 (58.5) | 0.93 |
| Age: 20-39 years old, n (%) | 9 (13) | 45 (13.4) | 0.97 |
| 40-59 years old, n (%) | 44 (63.8) | 217 (64.8) |  |
| ≥60 years old, n (%) | 16 (23.2) | 73 (21.8) |  |
| Mean age, years old (SD) | 55.8 (13.6) | 55.4 (13.5) | 0.82 |
| Follow up duration, mean years (SD) | 4.8 (3.1) | 5.9 (3.4) | 0.016 |
| Occupation: White collar, n (%) | 30 (43.5) | 154 (46) | 0.93 |
| Blue collar, n (%) | 26 (37.7) | 120 (35.8) |  |
| Other, n (%) | 13 (18.8) | 61 (18.2) |  |
| Urbanization (%) | 43 (62.3) | 240 (71.6) | 0.12 |
| Non-urbanization (%) | 26 (37.7) | 95 (28.4) |  |
| Month Income: 0–35000 NTD, n (%) | 57 (82.6) | 268 (80) | 0.62 |
| ≥35000 NTD, n (%) | 12 (17.4) | 67 (20) |  |
| Chronic kidney disease, n (%) | 48 (69.6) | 40 (11.9) | <0.001 |
| Nephrolithiasis, n (%) | 22 (31.9) | 19 (5.7) | <0.001 |
| Osteoporosis, n (%) | 6 (8.7) | 20 (6) | 0.4 |

Supplemental Table 2. The all death and cancer specific death, follow-up person-years and mortality rate. Estimated cox proportional hazard regression and 95% confidence intervals specified results of total and cancer-specific mortality.

|  | Total mortality | | | Cancer-specific mortality | | |
| --- | --- | --- | --- | --- | --- | --- |
|  | Control | PTC | Log-rank | Control | PTC | Log-rank |
| N | 360 | 72 |  | 360 | 72 |  |
| Event | 36 | 17 |  | 10 | 12 |  |
| Person-year | 2015 | 362.7 |  | 2015 | 362.7 |  |
| Incidence Rate (/1000 person-year) | 17.9 | 46.9 | <0.001 | 5 | 33.1 | <0.001 |
| Unadjusted | 1 | 5.98 (3.02-11.9) |  | 1 | 15.8 (5.09-49) |  |
| Model 1 | 1 | 5.83 (2.93-11.6) |  | 1 | 15.2 (4.88-47.2) |  |
| Model 2 | 1 | 6.06 (2.97-12.4) |  | 1 | 19.1 (5.91-61.9) |  |
| Model 3 | 1 | 3.49 (1.34-9.06) |  | 1 | 18.6 (4.2-82.4) |  |

Note: Model 1: adjusted for age and sex; Model 2: adjusted occupation, urbanization, average month income; Model 3: Model 2 additionally chronic kidney disease, nephrolithiasis, osteoporosis; Abbreviation: PTC: parathyroid cancer

Supplemental Table 3. Estimated sub-distribution competing hazard ratios for metabolic and heart comorbidities and mortality using multivariable regression model

(a) Metabolic comorbidities: Hypertension, diabetes mellitus and hyperlipidemia

|  | Hypertension | | Diabetes Mellitus | | Hyperlipidemia | |
| --- | --- | --- | --- | --- | --- | --- |
|  | Control | PTC | Control | PTC | Control | PTC |
| Unadjusted | 1 | 1.28 (0.50-3.28) | 1 | 9.19 (2.38-35.5) | 1 | 4.12 (1.39-12.2) |
| Model 1 | 1 | 1.34 (0.53-3.41) | 1 | 8.48 (2.16-33.3) | 1 | 4.03 (1.38-11.8) |
| Model 2 | 1 | 1.37 (0.51-3.63) | 1 | 9.19 (2.26-37.3) | 1 | 4.18 (1.39-12.6) |
| Model 3 | 1 | 1.10 (0.35-3.45) | 1 | 9.27 (1.35-63.8) | 1 | 5.54 (1.72-17.8) |

Note: Model 1: adjusted for age and sex; Model 2: adjusted occupation, urbanization, average month income; Model 3: Model 2 additionally chronic kidney disease, nephrolithiasis, osteoporosis; Abbreviation: PTC: parathyroid cancer

(B) Heart comorbidities: atrial fibrillation, coronary artery disease and heart failure

|  | Atrial fibrillation | | Coronary artery disease | | Heart failure | |
| --- | --- | --- | --- | --- | --- | --- |
|  | Control | PTC | Control | PTC | Control | PTC |
| Unadjusted | 1 | 3.19 (0.91-11.2) | 1 | 3.09 (1.55-9.82) | 1 | 4.19 (1.43-12.3) |
| Model 1 | 1 | 2.99 (0.82-11.0) | 1 | 3.57 (1.44-8.83) | 1 | 3.89 (1.30-11.7) |
| Model 2 | 1 | 2.94 (0.87-9.95) | 1 | 4.20 (1.66-10.6) | 1 | 4.18 (1.44-12.1) |
| Model 3 | 1 | 1.81 (0.60-5.46) | 1 | 1.48 (0.52-4.18) | 1 | 3.92 (1.21-12.6) |

Note: Model 1: adjusted for age and sex; Model 2: adjusted occupation, urbanization, average month income; M aTodel 3: Model 2 additionally chronic kidney disease, nephrolithiasis, osteoporosis; Abbreviation: PTC: parathyroid cancer

Supplemental table 4. Estimated Cox proportional hazard ration with 95% confidence interval stratified by time since diagnosis (years)

Supplemental Table 5 Association of adult parathyroid cancer compared matching general population with metabolic and heart comorbidities stratified by age less than 60 years old or older than 60 years old

|  | Age< 60 y/o | Age >= 60 | Interaction *P* |
| --- | --- | --- | --- |
| HTN | 1.12 (0.18-7.02) | 2.22 (0.38-13) | 0.14 |
| DM | 15.2 (1.00-231.2) | 22.5 (0.9-561.4) | 0.17 |
| HLY | 6.74 (1.26-36.2) | 12.1 (0.61-240.6) | 0.79 |
| Af | 2.34 (0.1-53.9) | 2.09 (0.36-12) | 0.43 |
| CAD | 0.33 (0.02-6.51) | 5.03 (0.94-27) | 0.012 |
| HF | 4.79 (0.52-44.2) | 3.73 (0.48-28.8) | 0.46 |

Supplemental table 6. The baseline characteristics of adult parathyroid carcinoma and all-covariate-matched controls without preexisting disease in the sensitivity analysis (a) hypertension cohort (b) diabetes cohort (c) hyperlipidemia cohort (d) atrial fibrillation cohort (e) coronary artery disease (f) heart failure.

1. Hypertension cohort

| Baseline Characteristics of adult parathyroid carcinoma and matching controls | | | |
| --- | --- | --- | --- |
|  | Control group (n=169) | Patients (n=41) | *P* value |
| Women, n (%) | 110 (65.1) | 26 (63.4) | 0.84 |
| Age: 20-39 years old, n (%) | 15 (8.9) | 3 (7.3) | 0.76 |
| 40-59 years old, n (%) | 125 (74) | 29 (70.7) |  |
| ≥60 years old, n (%) | 29 (17.2) | 9 (22) |  |
| Mean age, years old (SD) | 55 (11.6) | 56.5 (11.9) | 0.46 |
| Follow up duration, mean years (SD) | 5.4 (3.5) | 4.7 (3.5) | 0.2 |
| Occupation: White collar, n (%) | 13 (7.7) | 3 (7.3) | 0.99 |
| Blue collar, n (%) | 83 (49.1) | 20 (48.8) |  |
| Other, n (%) | 73 (43.2) | 18 (43.9) |  |
| Urbanization (%) | 94 (55.6) | 23 (56.1) | 0.96 |
| Non-urbanization (%) | 75 (44.4) | 18 (43.9) |  |
| Month Income: 0–35000 NTD, n (%) | 143 (84.6) | 34 (82.9) | 0.79 |
| ≥35000 NTD, n (%) | 26 (15.4) | 7 (17.1) |  |
| Chronic kidney disease, n (%) | 93 (55) | 25 (61) | 0.49 |
| Nephrolithiasis, n (%) | 39 (23.1) | 10 (24.4) | 0.86 |
| Osteoporosis, n (%) | 18 (10.7) | 4 (9.8) | 0.87 |

1. Diabetes mellitus cohort

| Baseline Characteristics of adult parathyroid carcinoma and matching controls | | | |
| --- | --- | --- | --- |
|  | Control group (n=214) | Patients (n=56) | *P* value |
| Women, n (%) | 127 (59.4) | 127 (59.4) | 0.84 |
| Age: 20-39 years old, n (%) | 30 (14) | 6 (10.7) | 0.76 |
| 40-59 years old, n (%) | 147 (68.7) | 37 (66.1) |  |
| ≥60 years old, n (%) | 37 (17.3) | 13 (23.2) |  |
| Mean age, years old (SD) | 53.9 (12.7) | 56.3 (13) | 0.21 |
| Follow up duration, mean years (SD) | 5.8 (3.5) | 4.8 (3.4) | 0.05 |
| Occupation: White collar, n (%) | 28 (13.1) | 10 (17.9) | 0.99 |
| Blue collar, n (%) | 101 (47.2) | 24 (42.9) |  |
| Other, n (%) | 85 (39.7) | 22 (39.3) |  |
| Urbanization (%) | 131 (61.2) | 34 (60.7) | 0.96 |
| Non-urbanization (%) | 83 (38.8) | 22 (39.3) |  |
| Month Income: 0–35000 NTD, n (%) | 181 (84.6) | 47 (83.9) | 0.79 |
| ≥35000 NTD, n (%) | 33 (15.4) | 9 (16.1) |  |
| Chronic kidney disease, n (%) | 126 (58.9) | 37 (66.1) | 0.49 |
| Nephrolithiasis, n (%) | 54 (25.2) | 15 (26.8) | 0.86 |
| Osteoporosis, n (%) | 11 (5.1) | 4 (7.1) | 0.87 |

1. Hyperlipidemia cohort

| Baseline Characteristics of adult parathyroid carcinoma and matching controls | | | |
| --- | --- | --- | --- |
|  | Control group (n=290) | Patients (n=63) | *P* value |
| Women, n (%) | 168 (57.9) | 37 (58.7) | 0.91 |
| Age: 20-39 years old, n (%) | 29 (10) | 6 (9.5) | 0.95 |
| 40-59 years old, n (%) | 193 (66.6) | 41 (65.1) |  |
| ≥60 years old, n (%) | 68 (23.5) | 16 (25.4) |  |
| Mean age, years old (SD) | 56.7 (12.8) | 57.3 (12.9) | 0.73 |
| Follow up duration, mean years (SD) | 5.4 (3.3) | 4.7 (3.2) | 0.11 |
| Occupation: White collar, n (%) | 40 (13.8) | 10 (15.9) | 0.91 |
| Blue collar, n (%) | 128 (44.1) | 27 (42.9) |  |
| Other, n (%) | 122 (42.1) | 26 (41.3) |  |
| Urbanization (%) | 173 (59.7) | 38 (60.3) | 0.92 |
| Non-urbanization (%) | 117 (40.3) | 25 (39.7) |  |
| Month Income: 0–35000 NTD, n (%) | 245 (84.5) | 53 (84.1) | 0.94 |
| ≥35000 NTD, n (%) | 45 (15.5) | 10 (15.9) |  |
| Chronic kidney disease, n (%) | 196 (67.6) | 44 (69.8) | 0.73 |
| Nephrolithiasis, n (%) | 82 (28.3) | 18 (28.6) | 0.96 |
| Osteoporosis, n (%) | 25 (8.6) | 6 (9.5) | 0.82 |

1. Atrial fibrillation cohort

| Baseline Characteristics of adult parathyroid carcinoma and matching controls | | | |
| --- | --- | --- | --- |
|  | Control group (n=307) | Patients (n=64) | *P* value |
| Women, n (%) | 185 (60.3) | 39 (60.9) | 0.92 |
| Age: 20-39 years old, n (%) | 30 (9.8) | 6 (9.4) | 0.98 |
| 40-59 years old, n (%) | 199 (64.8) | 41 (64.1) |  |
| ≥60 years old, n (%) | 78 (25.4) | 17 (26.6) |  |
| Mean age, years old (SD) | 56.8 (12.8) | 57.1 (12.9) | 0.84 |
| Follow up duration, mean years (SD) | 5.5 (3.5) | 4.5 (3.1) | 0.06 |
| Occupation: White collar, n (%) | 53 (17.3) | 12 (18.8) | 0.95 |
| Blue collar, n (%) | 134 (43.7) | 27 (42.2) |  |
| Other, n (%) | 120 (39.1) | 25 (39.1) |  |
| Urbanization (%) | 191 (62.2) | 40 (62.5) | 0.97 |
| Non-urbanization (%) | 116 (37.8) | 24 (37.5) |  |
| Month Income: 0–35000 NTD, n (%) | 260 (84.7) | 54 (84.4) | 0.95 |
| ≥35000 NTD, n (%) | 47 (15.3) | 10 (15.6) |  |
| Chronic kidney disease, n (%) | 208 (67.8) | 44 (68.8) | 0.88 |
| Nephrolithiasis, n (%) | 86 (28) | 18 (28.1) | 0.99 |
| Osteoporosis, n (%) | 27 (8.8) | 6 (9.4) | 0.88 |

1. Coronary artery disease

| Baseline Characteristics of adult parathyroid carcinoma and matching controls | | | |
| --- | --- | --- | --- |
|  | Control group (n=287) | Patients (n=63) | *P* value |
| Women, n (%) | 173 (60.3) | 173 (60.3) | 1 |
| Age: 20-39 years old, n (%) | 30 (10.5) | 6 (9.5) | 0.89 |
| 40-59 years old, n (%) | 192 (66.9) | 41 (65.1) |  |
| ≥60 years old, n (%) | 65 (22.7) | 16 (25.4) |  |
| Mean age, years old (SD) | 56 (12.8) | 57 (13) | 0.6 |
| Follow up duration, mean years (SD) | 5.6 (3.5) | 4.8 (3.1) | 0.09 |
| Occupation: White collar, n (%) | 44 (15.3) | 11 (17.5) | 0.91 |
| Blue collar, n (%) | 132 (46) | 28 (44.4) |  |
| Other, n (%) | 111 (38.7) | 24 (38.1) |  |
| Urbanization (%) | 172 (59.9) | 38 (60.3) | 0.95 |
| Non-urbanization (%) | 115 (40.1) | 25 (39.7) |  |
| Month Income: 0–35000 NTD, n (%) | 237 (82.6) | 52 (82.5) | 0.99 |
| ≥35000 NTD, n (%) | 50 (17.4) | 11 (17.5) |  |
| Chronic kidney disease, n (%) | 188 (65.5) | 43 (68.3) | 0.68 |
| Nephrolithiasis, n (%) | 71 (24.7) | 16 (25.4) | 0.91 |
| Osteoporosis, n (%) | 20 (7) | 5 (7.9) | 0.79 |

1. Heart failure

| Baseline Characteristics of adult parathyroid carcinoma and matching controls | | | |
| --- | --- | --- | --- |
|  | Control group (n=247) | Patients (n=58) | *P* value |
| Women, n (%) | 150 (60.7) | 36 (62.1) | 0.85 |
| Age: 20-39 years old, n (%) | 25 (10.1) | 5 (8.6) | 0.85 |
| 40-59 years old, n (%) | 166 (67.2) | 38 (65.5) |  |
| ≥60 years old, n (%) | 56 (22.7) | 15 (25.9) |  |
| Mean age, years old (SD) | 55.5 (12.4) | 56.8 (12.6) | 0.46 |
| Follow up duration, mean years (SD) | 5.5 (3.4) | 4.8 (3.2) | 0.19 |
| Occupation: White collar, n (%) | 30 (12.2) | 8 (13.8) | 0.9 |
| Blue collar, n (%) | 116 (47) | 25 (43.1) |  |
| Other, n (%) | 101 (40.9) | 25 (43.1) |  |
| Urbanization (%) | 164 (66.4) | 37 (63.8) | 0.71 |
| Non-urbanization (%) | 83 (33.6) | 21 (36.2) |  |
| Month Income: 0–35000 NTD, n (%) | 211 (85.4) | 49 (84.5) | 0.86 |
| ≥35000 NTD, n (%) | 36 (14.6) | 9 (15.5) |  |
| Chronic kidney disease, n (%) | 157 (63.6) | 39 (67.2) | 0.6 |
| Nephrolithiasis, n (%) | 69 (27.9) | 16 (27.6) | 0.96 |
| Osteoporosis, n (%) | 18 (7.3) | 6 (10.3) | 0.44 |

Supplemental table 7. The incidence case, follow-up person-years and rate of individual metabolic and heart comorbidities and estimated cox proportional hazard regression and 95% confidence intervals specified results of hypertension, diabetes mellitus, hyperlipidemia, atrial fibrillation, coronary artery disease and heart failure in the sensitivity analysis for all-covariate-matching parathyroid cancer population.

1. Metabolic comorbidities: hypertension, diabetes mellitus and hyperlipidemia

|  | Hypertension | | | Diabetes | | | Hyperlipidemia | | |
| --- | --- | --- | --- | --- | --- | --- | --- | --- | --- |
|  | Control | PTC | Log-rank | Control | PTC | Log-rank | Control | PTC | Log-rank |
| N | 169 | 41 |  | 214 | 56 |  | 290 | 63 |  |
| Event | 17 | 5 |  | 7 | 6 |  | 19 | 5 |  |
| Person-year | 839 | 190.9 |  | 1149 | 268.1 |  | 1397 | 296.4 |  |
| Incidence Rate (/1000 person-year) | 20.3 | 26.19 | 0.5 | 6.09 | 22.38 | 0.07 | 13.6 | 16.87 | 0.59 |
| Unadjusted | 1 | 1.41 (0.52-3.82) |  | 1 | 2.41 (0.89-6.52) |  | 1 | 1.31 (0.49-3.52) |  |

Note: PTC: parathyroid cancer

(b) Heart comorbidities: atrial fibrillation, coronary artery disease and heart failure

|  | Atrial fibrillation | | | Coronary artery disease | | | Heart failure | | |
| --- | --- | --- | --- | --- | --- | --- | --- | --- | --- |
|  | Control | PTC | Log-rank | Control | PTC | Log-rank | Control | PTC | Log-rank |
| N | 307 | 64 |  | 287 | 63 |  | 247 | 58 |  |
| Event | 6 | 4 |  | 9 | 7 |  | 13 | 5 |  |
| Person-year | 1515 | 291.2 |  | 1439 | 300.1 |  | 1249 | 277.8 |  |
| Incidence Rate (/1000 person-year) | 3.96 | 13.74 | 0.001 | 6.26 | 23.33 | 0.003 | 10.4 | 18 | 0.27 |
| Unadjusted | 1 | 10.05 (1.84-54.99) |  | 1 | 3.64 (1.48-8.96) |  | 1 | 1.77 (0.64-4.93) |  |

Note: PTC: parathyroid cancer

Supplement Figure 1, Flowchart of Patients with parathyroid cancer in National Taiwan Cancer Registry Database cohort from 2007 to 2018 who met Inclusion and Exclusion Criteria


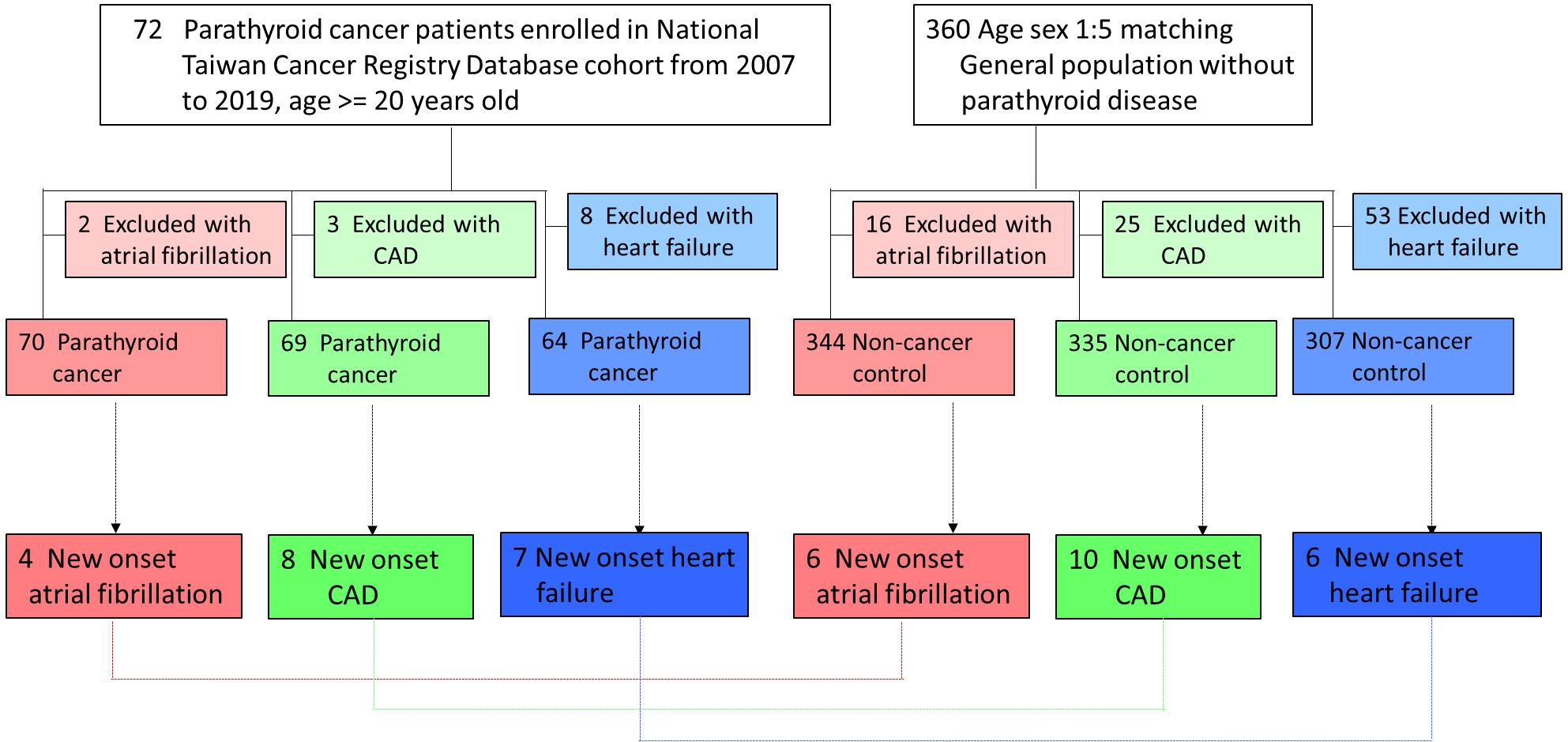


Supplement Figure 2. The Kaplan-Meier of overall survival, hypertension and atrial fibrillation cases among adult parathyroid cancer compared with matching general

1. Overall survival

population
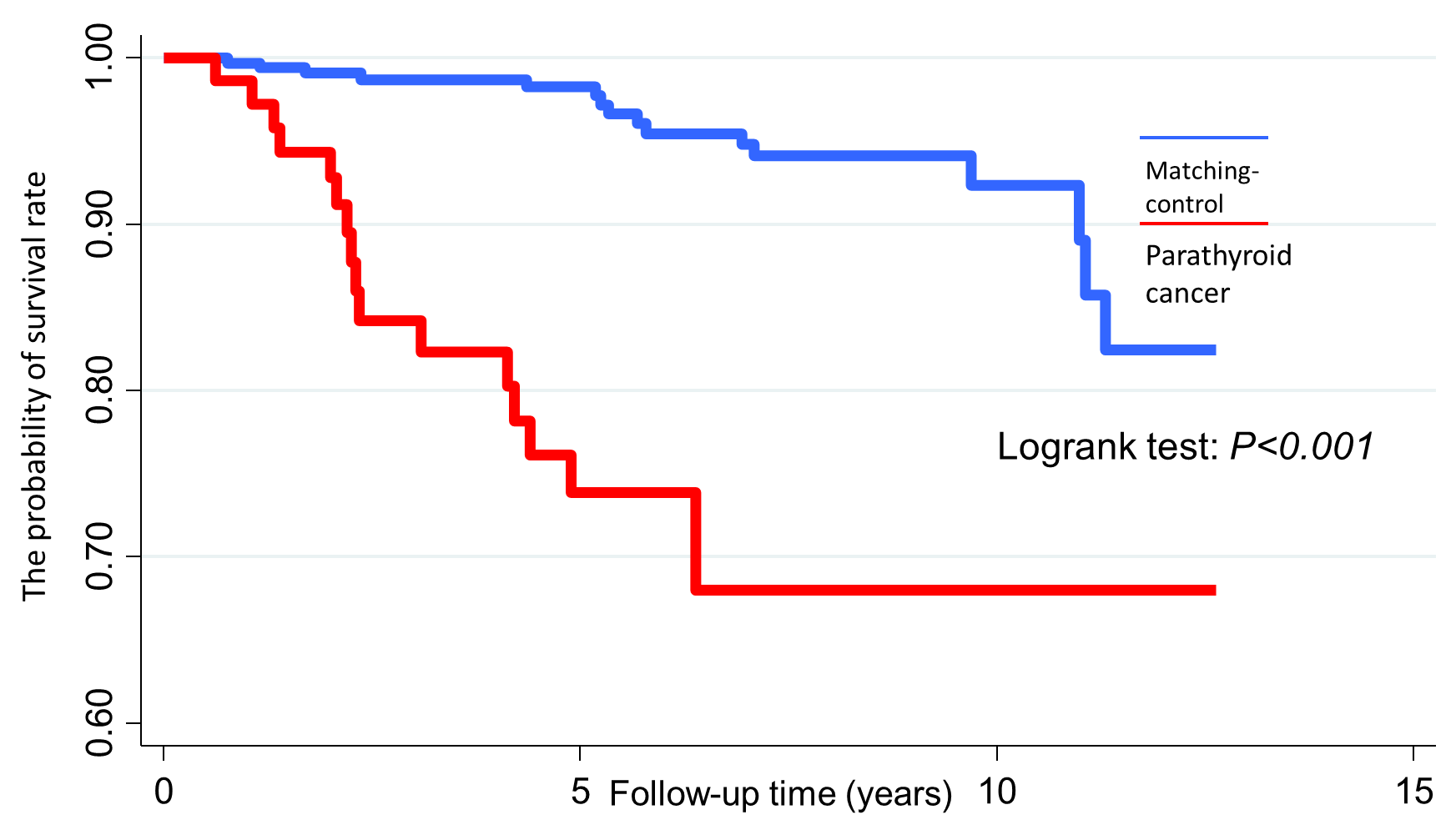


1. Hypertension
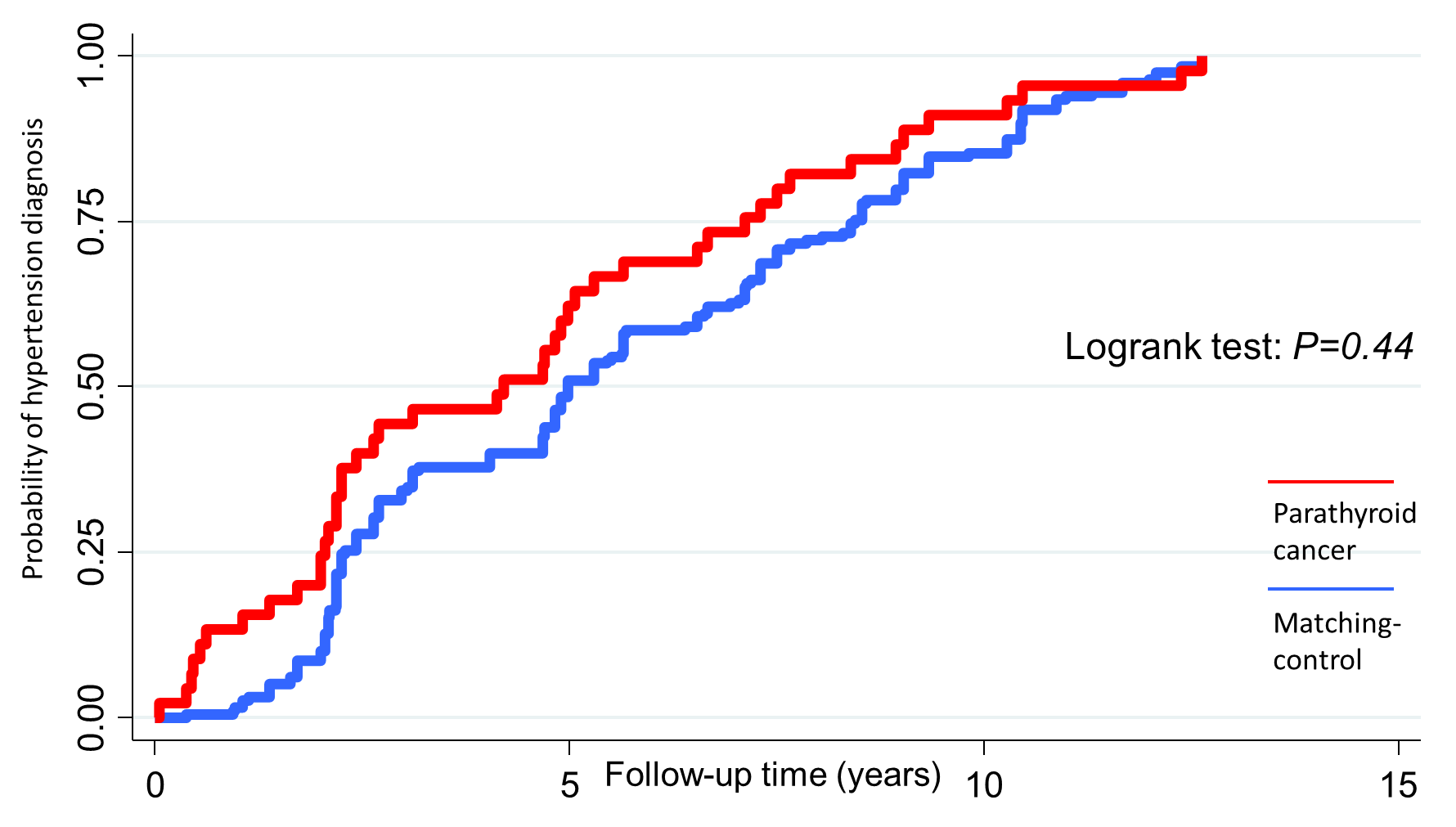

2. Atrial fibrillation


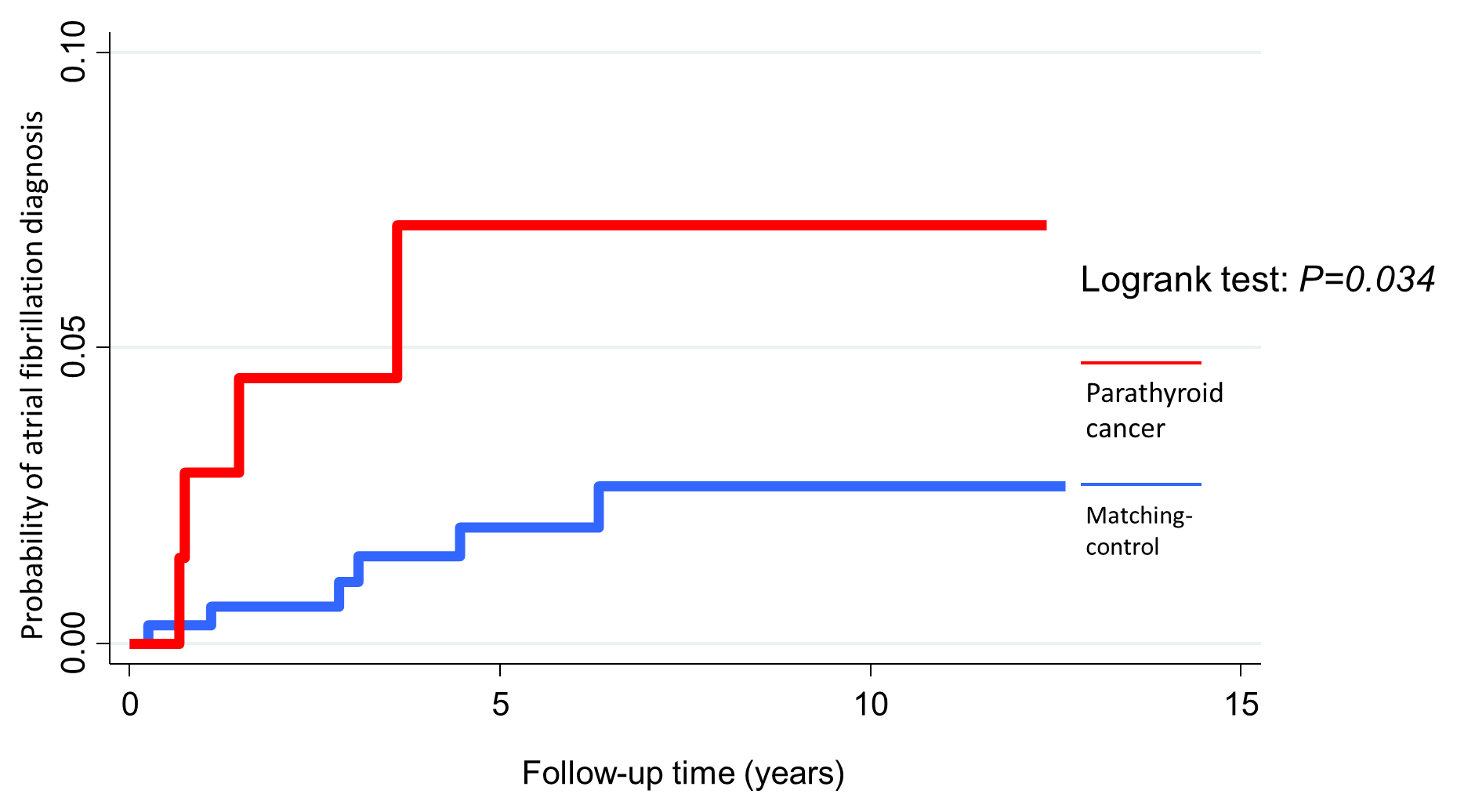


Supplement Figure 3. Estimated the cumulative incidence of metabolic and heart comorbidities among adult parathyroid cancer compared with general population. (a) Hypertension (b) diabetes mellitus (c) hyperlipidemia (e) atrial fibrillation (f) coronary artery disease (g) heart failure

(a) Hypertension (HTN)


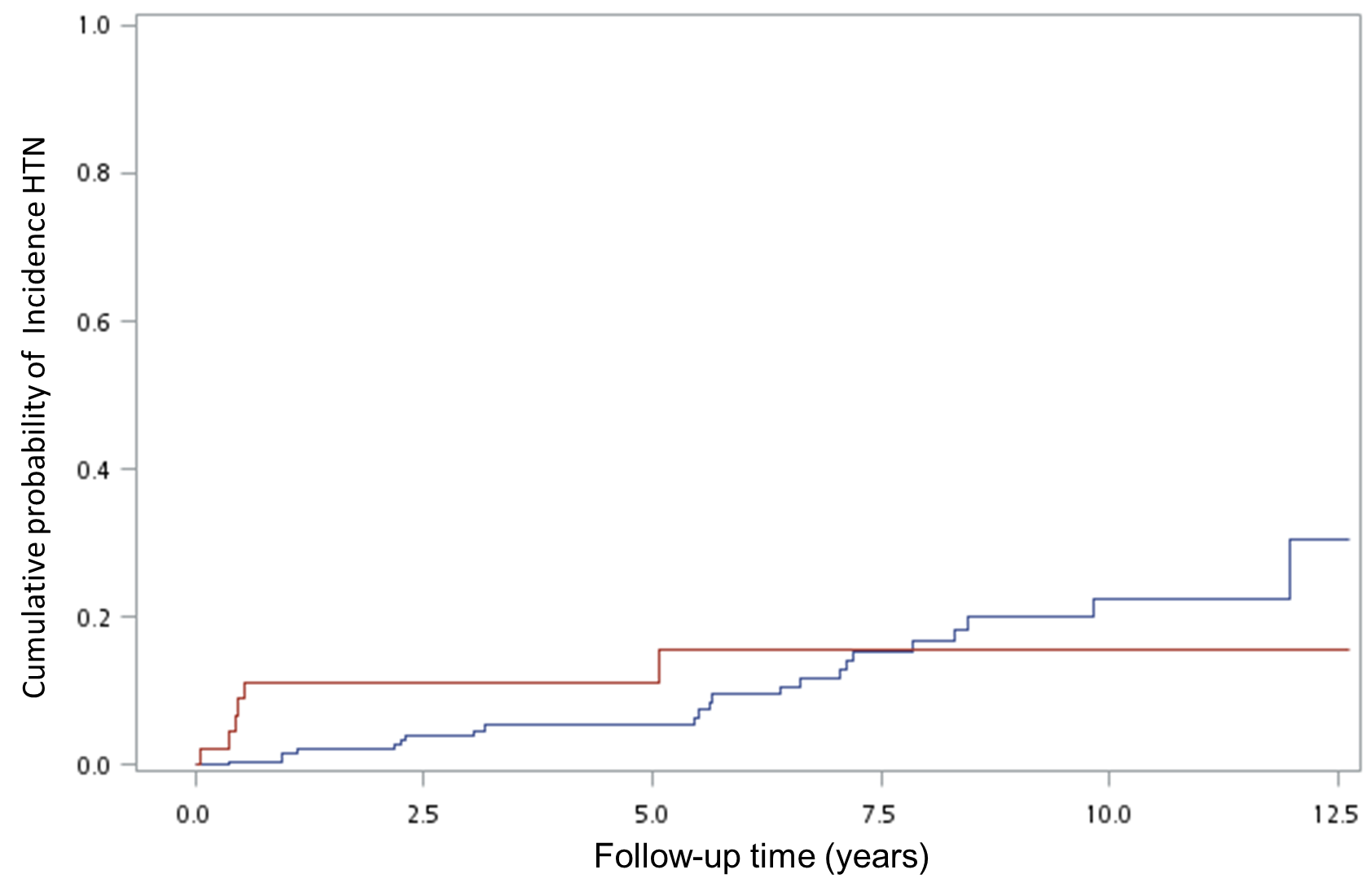


(b) Diabetes Mellitus (DM)


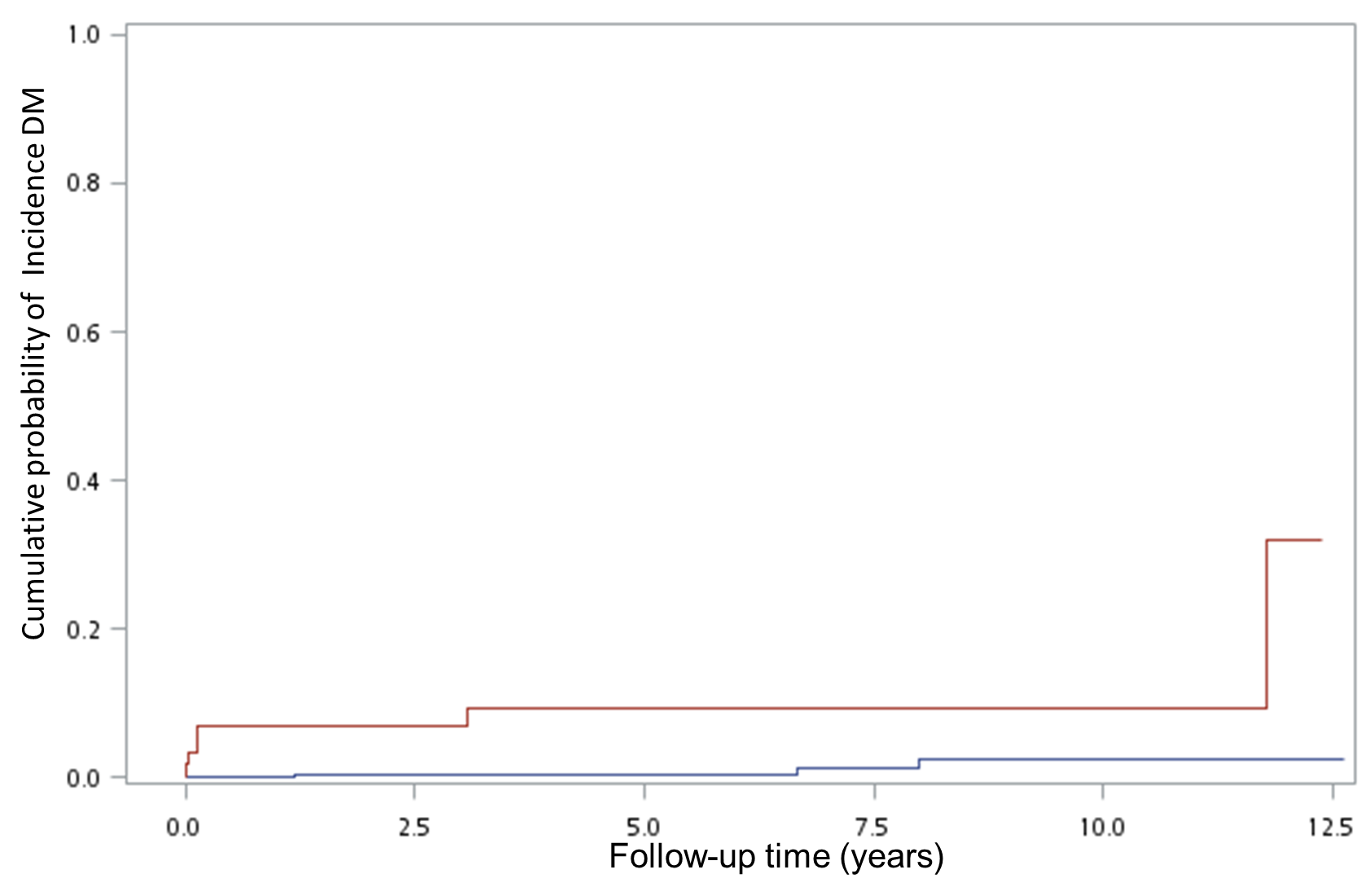


(C) Hyperlipidemia (HLY)


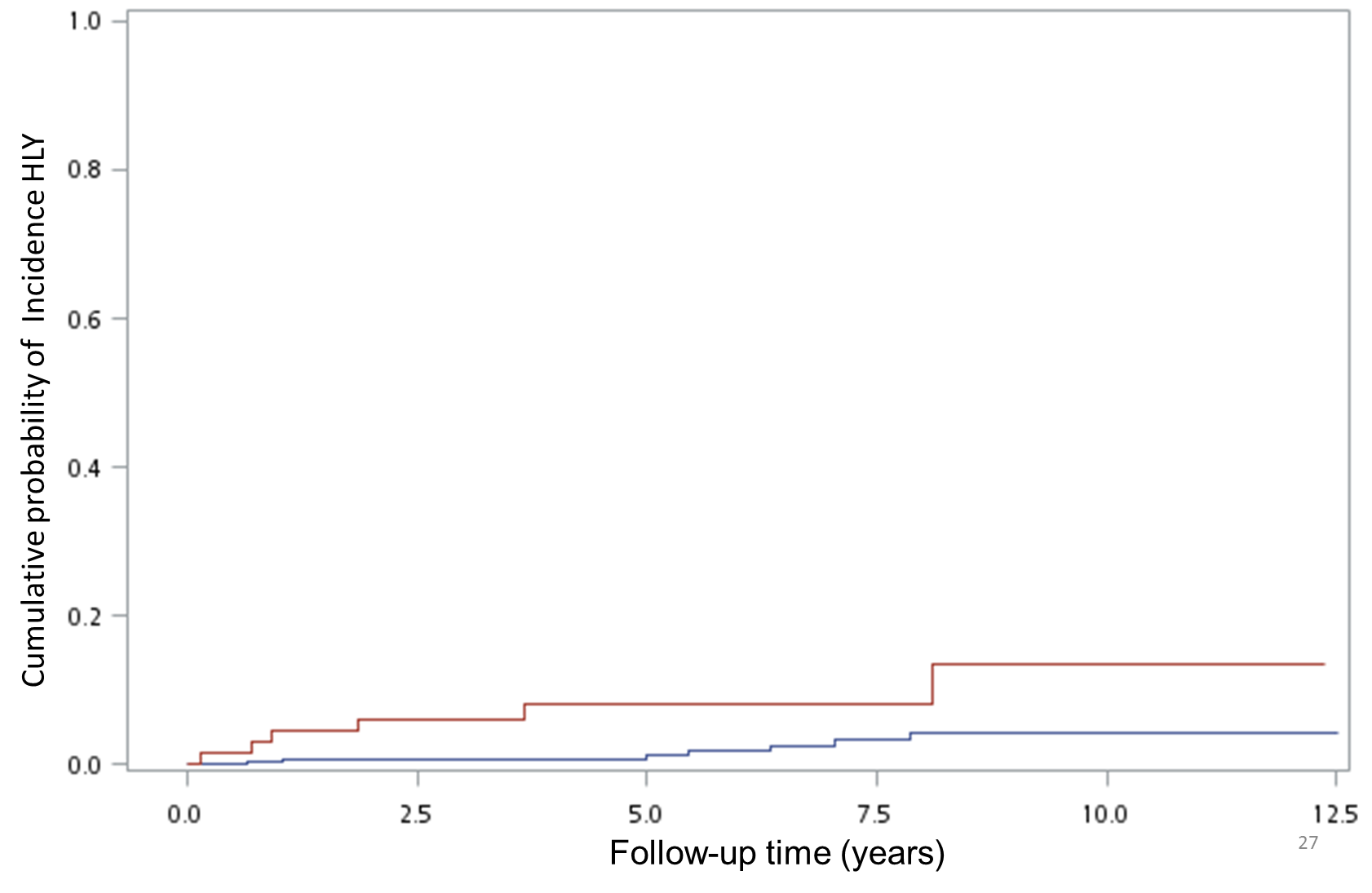


(d) Atrial fibrillation (Af)


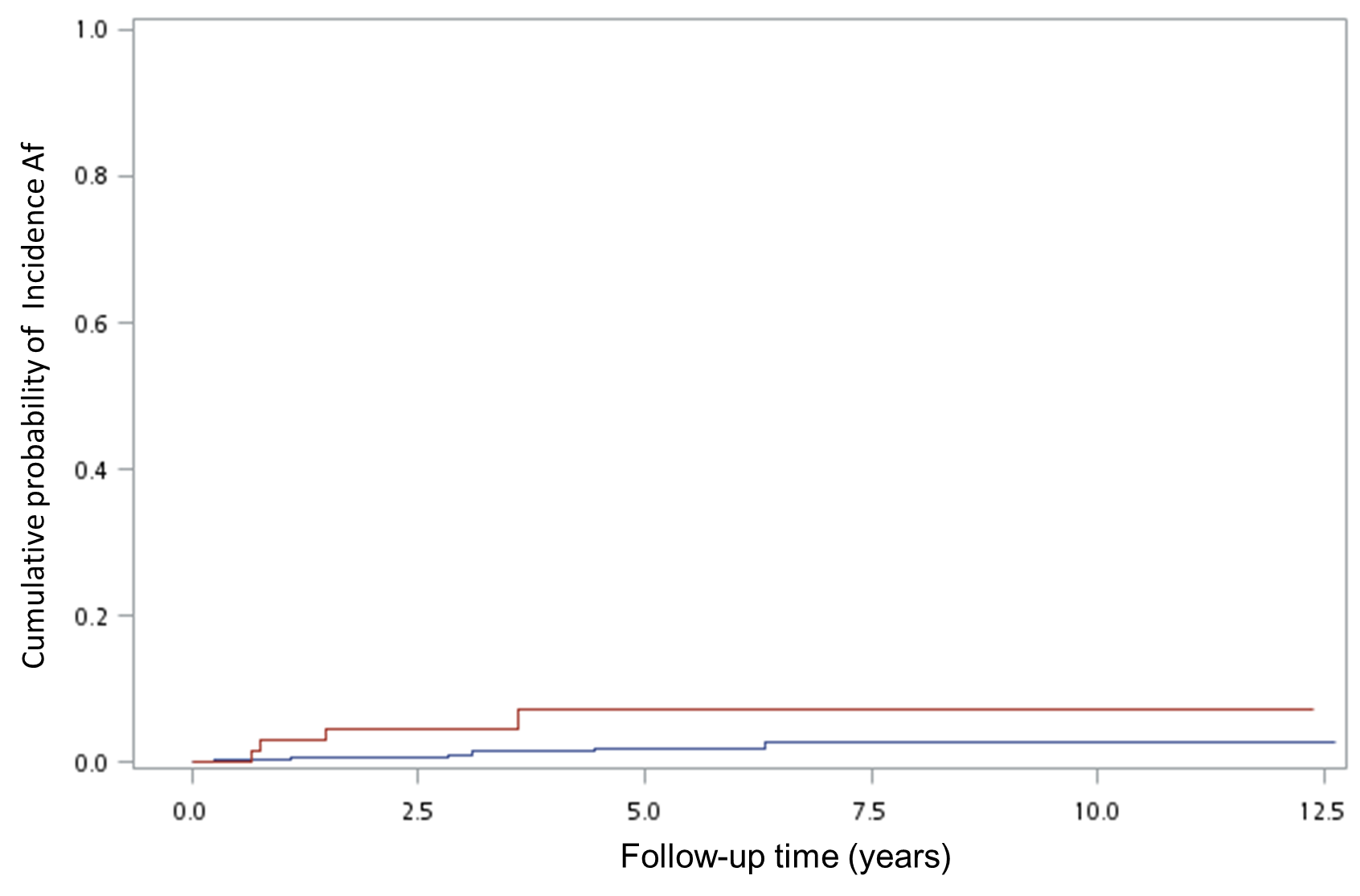


(e) coronary artery disease (CAD)


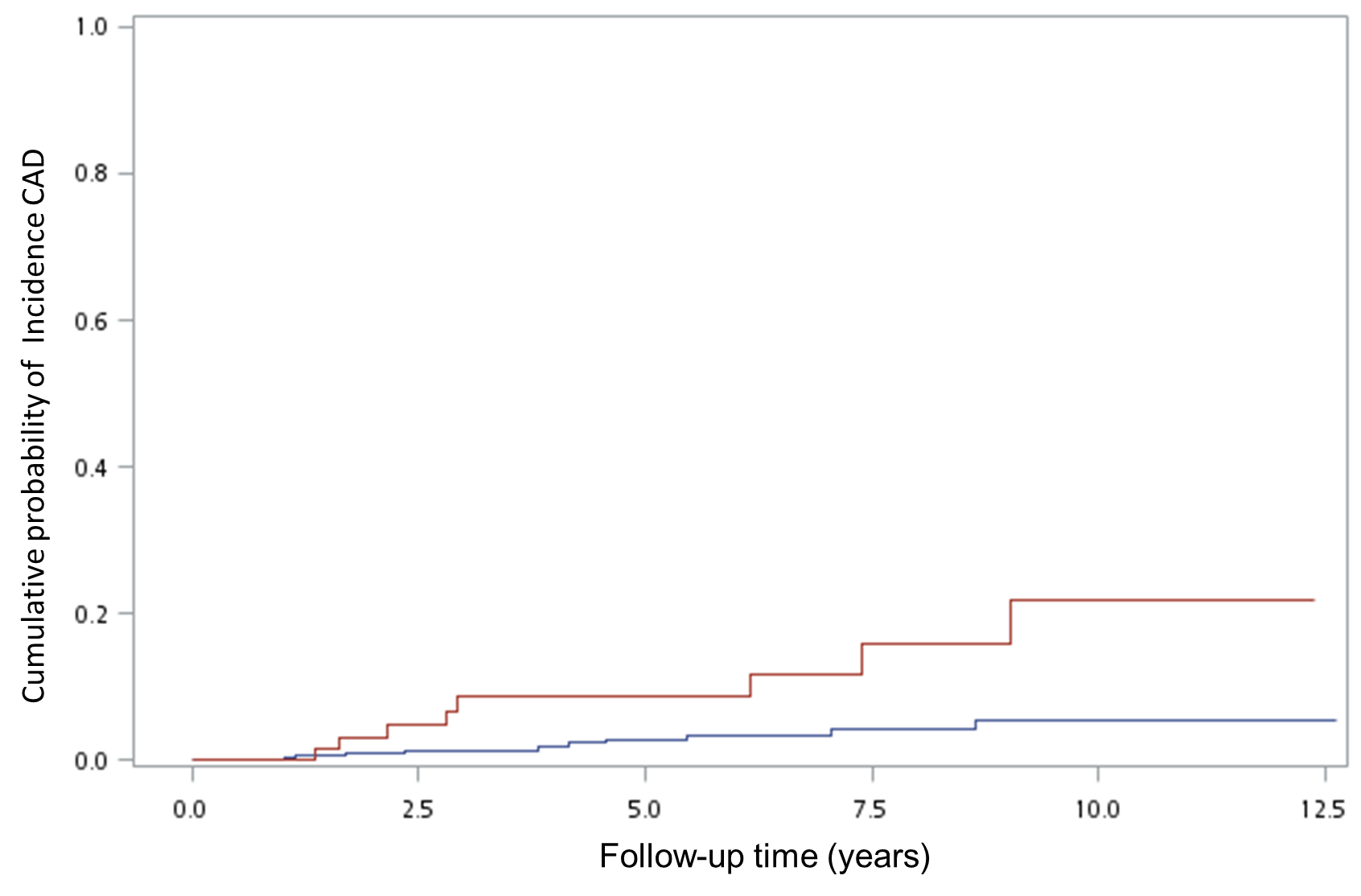


(e) Heart failure (HF)


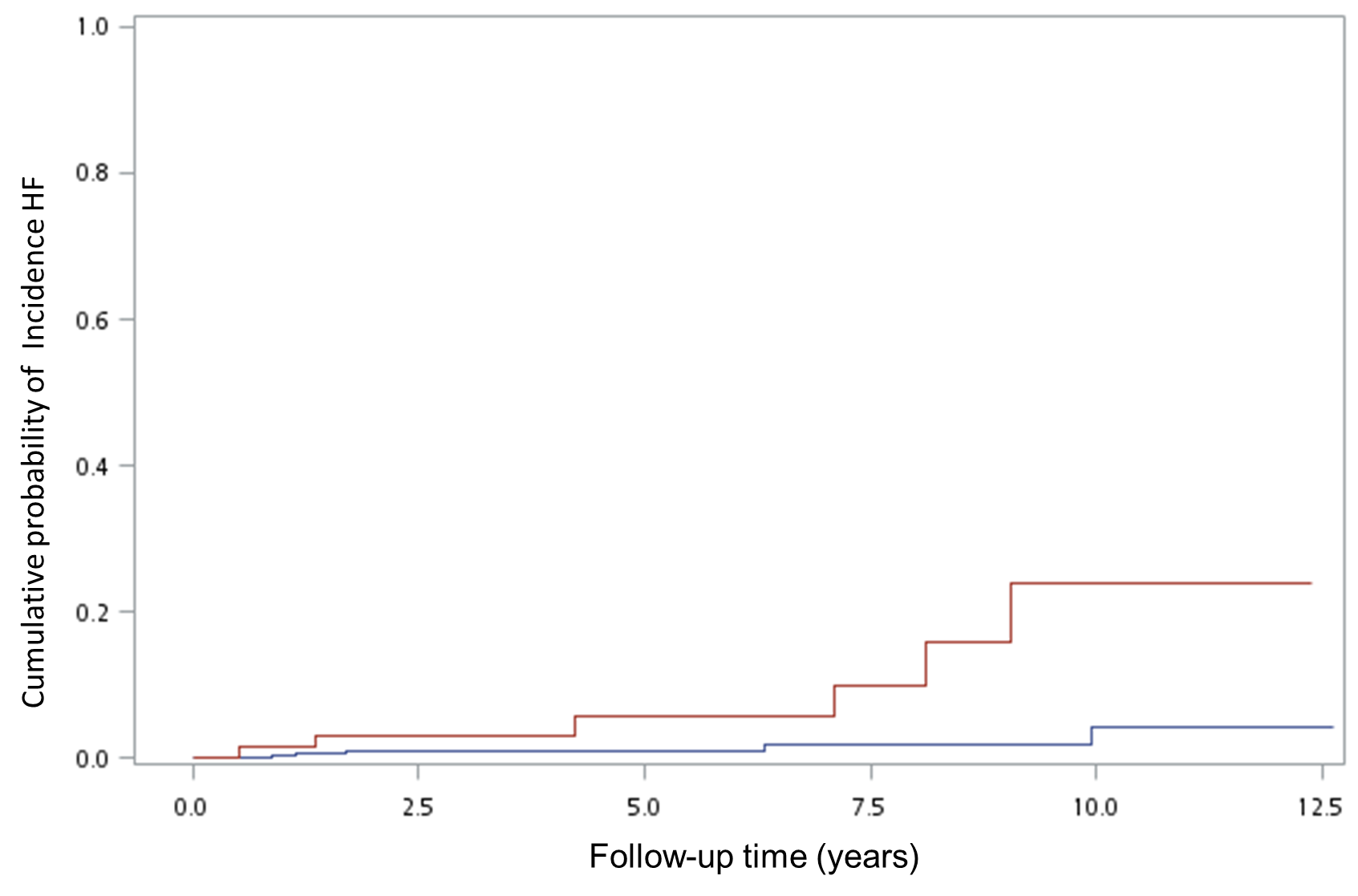


Supplement Figure 4. Association of adult parathyroid cancer compared matching general population with metabolic and heart comorbidities stratified by age less than 60 years old or older than 60 years old


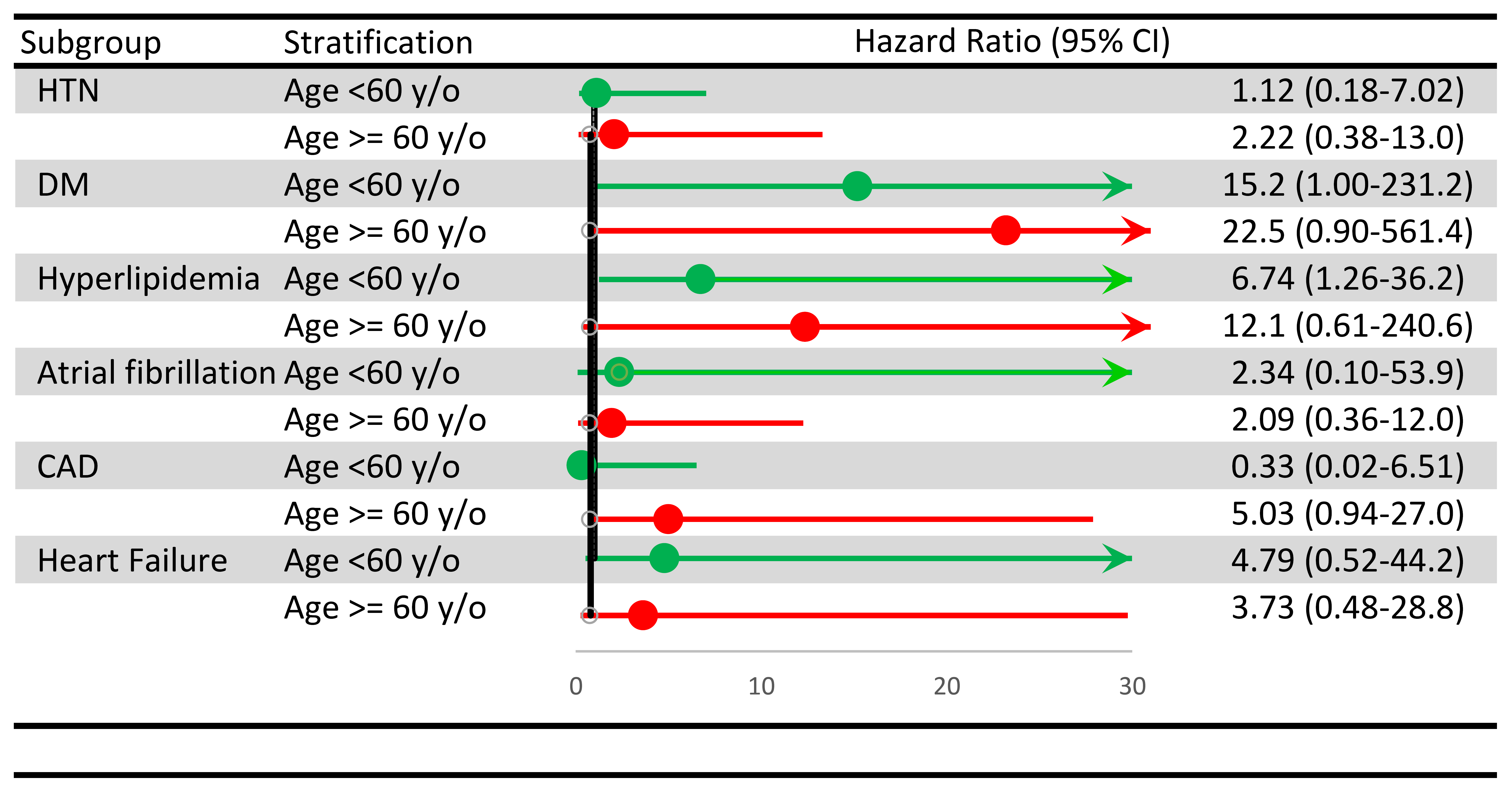


Supplement Figure 5. The log(-log(survival time)) versus log of metabolic and heart comorbidities event-free survival time including time independent covariates (A) hypertension (b)diabetes mellitus (c) hyperlipidemia (d) atrial fibrillation (e) coronary artery disease (f) heart failure

(a) Hypertension


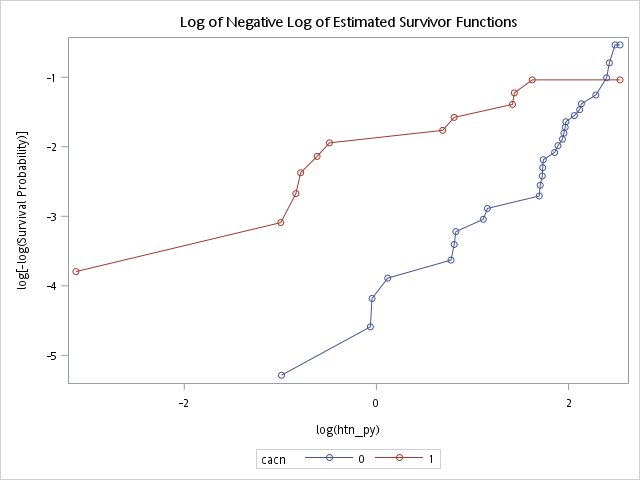


(b) Diabetes mellitus


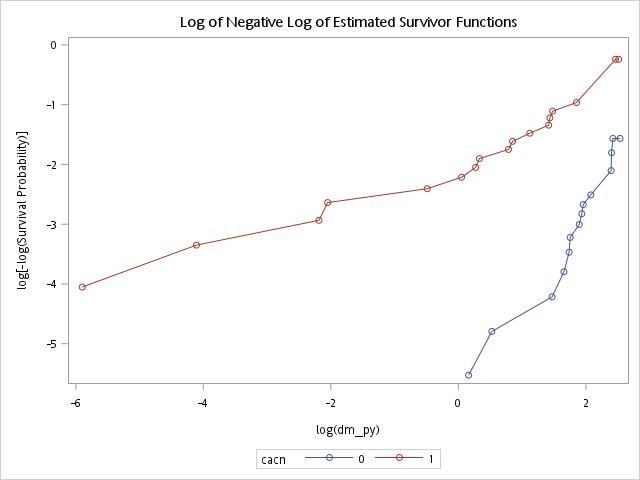


(c) Hyperlipidemia


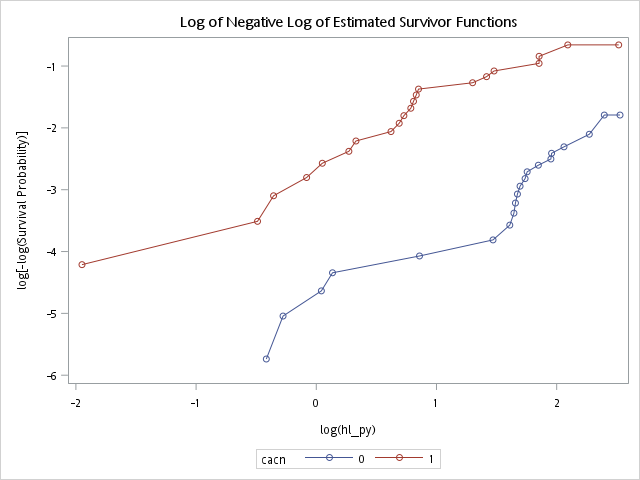


(d) atrial fibrillation


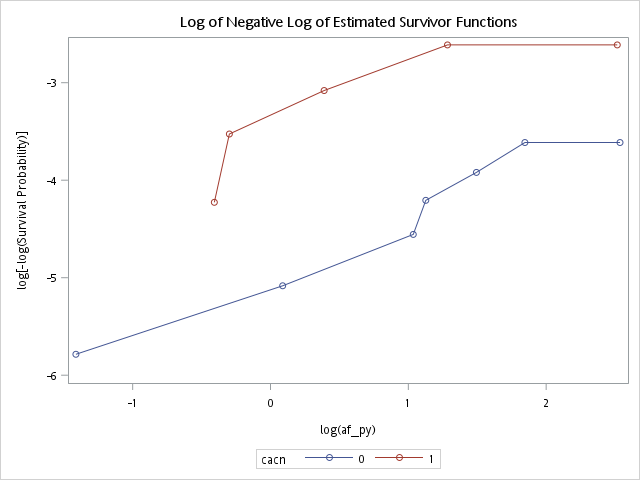


(e) coronary artery disease


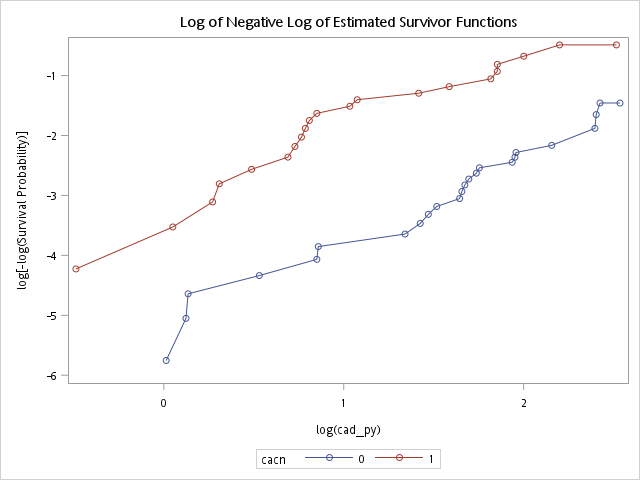


(f) heart failure


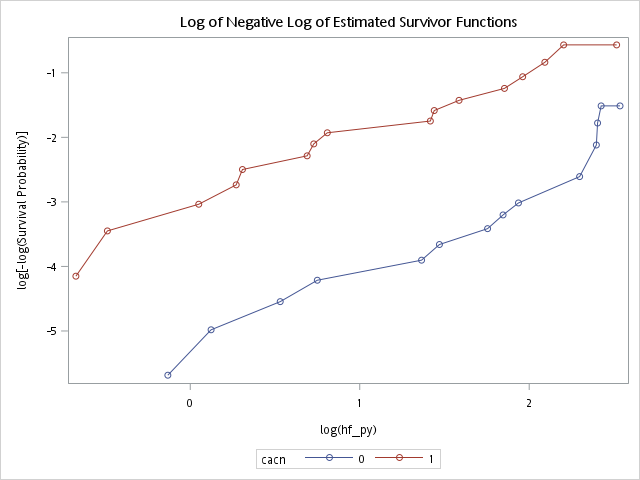

Supplement: Supplementary file 1 — Additional file 1: Tables S1. Baseline Characteristics. Tables S2. Cox proportional hazard regression of total and cancer-specific mortality. Tables S3. Estimated sub-distribution competing hazard ratios for metabolic and heart comorbidities and mortality. Tables S4. Estimated Cox proportional hazard ration with 95% confidence interval stratified by time since diagnosis. Tables S5. Metabolic and heart comorbidities stratified by age less than 60 years old or older than 60 years old. Tables S6. Sensitivity analysis (a) hypertension cohort (b) diabetes cohort (c) hyperlipidemia cohort (d) atrial fibrillation cohort (e) coronary artery disease (f) heart failure. Tables S7. The sensitivity analysis for all-covariate-matching parathyroid cancer population. Figures S1. Flowchart of Patients with parathyroid cancer. Figures S2. The Kaplan-Meier of overall survival, hypertension and atrial fibrillation. Figures S3. The cumulative incidence of metabolic and heart comorbiditiesHypertensiondiabetes mellitushyperlipidemiaatrial fibrillationcoronary artery diseaseheart failure. Figures S4. Association of adult parathyroid cancer stratified by age less than 60 years old or older than 60 years old. Figures S5. The log) versus loghypertensiondiabetes mellitushyperlipidemiaatrial fibrillationcoronary artery diseaseheart failure. [file 12916_2023_2946_MOESM1_ESM.docx]
